# “My Sensory Experiences Tool”: A Neurodiversity‐Affirming Therapeutic Tool to Support the Sensory Challenges and Preferences of Autistic Children and Adults

**DOI:** 10.1155/oti/4779496

**Published:** 2026-02-25

**Authors:** Jill Ashburner, Victoria Tomkins, Cerys Downing, Emily Rietberg, Jessica Hill, Jodie Copley, Natasha Bobir

**Affiliations:** ^1^ Autism Queensland Limited, Sunnybank, Australia; ^2^ The School of Health and Rehabilitation Sciences, The University of Queensland, St Lucia, Queensland, Australia, uq.edu.au

## Abstract

**Background:**

My Sensory Experiences Tool (MYSET) is a picture‐based card‐sort tool designed to support conversations with autistic people about their sensory experiences with a view to enabling better understanding and accommodation of their sensory challenges.

**Purpose:**

This study aimed firstly to describe MYSET and the considerations that guided the development of the tool, and secondly to explore the perceptions of autistic people, family members and professional practitioners of the usefulness of MYSET and ways it could be improved.

**Method:**

We gathered the perspectives of 18 professional practitioners, five autistic individuals and four family members through semi‐structured interviews and focus groups. The data was analysed through inductive content analysis.

**Findings:**

The participants perceived that MYSET enabled the gathering of individualised qualitative information about the person’s sensory experiences. MYSET was also perceived to be accessible, including people ranging in age from 5 years to adulthood and people with abilities ranging from mild intellectual disability to average/high IQ. The tool facilitates conversations about the links between the person’s sensory responses and their daily life experiences. A key perceived outcome of MYSET was the enhancement of others’ capacity to understand and accommodate the autistic person’s sensory challenges. The tool was refined in response to participant feedback.

**Conclusion:**

MYSET enables the gathering of detailed, individualised qualitative data on the sensory experiences of an autistic person and the collaborative design of accommodations that are compatible with their lifestyle.

## 1. Introduction

The sensory experiences of autistic people are commonly reported to substantially impact their daily lives [1, 2]. Sensory experiences are private, internal events that are often unobservable by others [3]. Consequently, the sensory experiences of autistic people may often be overlooked or misunderstood, as articulated by Lawson [4]:

*Many a time, autistic individuals have been pushed beyond the limits of sensory endurance. Often this is due to those relating to them not having understood how painful it is to be overloaded by too much sound, and visual stimulation*.

Understanding the impact of sensory experiences on an autistic person’s life allows for greater understanding, compassion and advocacy, and can facilitate person‐centred planning and support.

This paper first describes an innovative therapeutic tool called the My Sensory Experiences Tool (MYSET), which aims to provide an accessible way for autistic people to explain their experiences of their sensory world. The considerations that guided the development of the tool are also outlined. Secondly, the paper explores the perceptions of autistic people, their family members and professional practitioners of the usefulness of MYSET and ways it could be improved.

Currently, the design of support strategies for autistic people who experience sensory challenges is typically based on standardised sensory processing assessments, interviews of family members and teachers, and observations [5]. These processes, however, have some limitations that can diminish the quality of information gathered and, in turn, the quality of support strategies developed for autistic people.

First, the voice of the autistic person is often missing from the information gathering process. Information on the sensory differences of autistic children is most often gathered using proxy‐report questionnaires completed by parents or teachers. The most frequently used sensory processing assessments for children are the Sensory Profile [6] and the Sensory Processing Measure [7], as these assessments are commercially available and are therefore readily accessible to clinicians. Although other sensory assessments for children are used in research, they are not easily accessed by clinicians [8]. While the input of parents is undoubtedly valuable, many parents express concern about being unsure of how their child experiences sensations [9].

Sensory processing self‐report measures for adolescents and adults that are readily available to clinicians include the Adolescent/Adult Sensory Profile [10] and the Glasgow Sensory Questionnaire (Roberts & Simmons, 2023). The complexity of the language involved in these self‐report measures reduces their accessibility to a substantial proportion of autistic people, particularly those who experience communication differences [11, 12].

The quality of information gathered through observation of sensory experiences may also often be limited. Observations are interpretations of behaviours that may or may not accurately reflect what the autistic person is experiencing [3]. When we observe a person in a real‐life setting, there may be multiple possible explanations for their behaviour (e.g., a student withdrawing from a busy school playground may be responding to excessive noise or tactile input, or alternatively to the fear of being bullied by other students). Sensory experiences set up in a clinic setting for the purposes of observation do not reflect real‐life environments, which are typically characterised by multi‐sensory and unpredictable input [13]. For example, many autistic people cope well when presented with a single sound, light, or piece of fabric in a controlled environment because the sensory stimulus is predictable, and one sense is presented at a time. They may, however, become highly distressed in a busy food hall, with lots of noise, bright lights, smells and movement of people [13].

The sensory responses of autistic people are known to be highly heterogenous, with frequent reports of variable and, at times, opposite responses [14]. For example, some autistic people report reduced awareness of pain, while others report heightened awareness of pain [15]. Standardised sensory processing assessments, however, assume that participants are likely to report reduced awareness of pain and therefore preclude the reporting of heightened pain awareness. For example, the Glasgow Sensory Questionnaire [16] asks adults to rate the frequency with which they “*notice that they have hurt themselves but do not feel any pain*.” One‐size‐fits‐all standardised sensory questionnaires may therefore not fully capture the idiosyncratic sensory experiences of autistic people.

Lucas et al. [17] observes that most children’s sensory assessments focus primarily on the sensory systems, with minimal emphasis on daily life participation or the impact on families. Consequently, clinicians may struggle to link quantitative sensory processing scores with a plan to support the person’s daily routines. For example, a standardised sensory processing assessment may indicate tactile sensitivity but may not elicit information on the person’s reactions to being touched by hairdressers, doctors or dentists. It is important that clinicians understand and address these barriers to participation.

A study by [1] highlighted important contextual factors that influence the sensory responses of autistic people. These factors include the person’s level of control, which affects the predictability of the sensory input (e.g., the sound of a blender used by others may be intolerable, but tolerable if the person is operating the blender himself as the sound is under his control). The sensory responses of autistic people may also be influenced by their current mood and energy levels (i.e., their sensory experiences may be more distressing if they are stressed or tired). The efficacy of their strategies to manage their sensory experiences may also have an impact (e.g., whether they are able to avoid overwhelming environments such as supermarkets by online shopping). The extent to which others in their lives understand their sensory needs can also mediate their capacity to cope with sensory input (e.g., others can help them navigate or avoid environments with overwhelming sensory input) [1]. Standardised sensory questionnaires are unlikely to elicit information on the impact of these contextual factors that are important for clinicians to understand when designing effective accommodations. For example, a standardised sensory processing assessment may indicate auditory sensitivity but may not reveal that the person’s negative reactions to noisy appliances such as vacuum cleaners only occur when they are turned on unexpectedly. If this information is shared with family members, they will understand the need to forewarn the person before using noisy appliances.

In summary, we identified several gaps in current sensory processing information‐gathering approaches including the following: (a) insufficient representation of the voice of autistic people, (b) lack of accessibility to people with communication differences, (c) observations that may not accurately reflect what the person is actually experiencing, (d) lack of capacity to accommodate the heterogeneity of the sensory experiences of autistic individuals, (e) insufficient exploration of the impact of sensory responses on daily life participation and (f) insufficient exploration of the influence of contextual factors.

DuBois et al. [8] suggested that semi‐structured interviews with the autistic person themselves (rather than just their caregivers) may be a useful alternative to standardised sensory processing assessments. As this approach had the potential to overcome the gaps in current sensory processing information‐gathering approaches that are summarised above, it influenced the design of MYSET. By asking autistic people directly, we can give voice to their concerns about the impact of sensory experiences on their daily lives. Open‐ended questions can be used flexibly to gain an understanding of the person’s unique daily life experiences, and the contextual factors that are relevant to their lives. DuBois et al. [8] recommend using visual cues to accommodate different communication capabilities during these interviews. Accordingly, MYSET was designed to facilitate semi‐structured conversations using picture‐based card sorting.

## 2. Materials and Methods

The processes involved in the development of MYSET, the factors that guided its development and a description of MYSET are outlined below.

### 2.1. Development of MYSET

**Figure 1:**
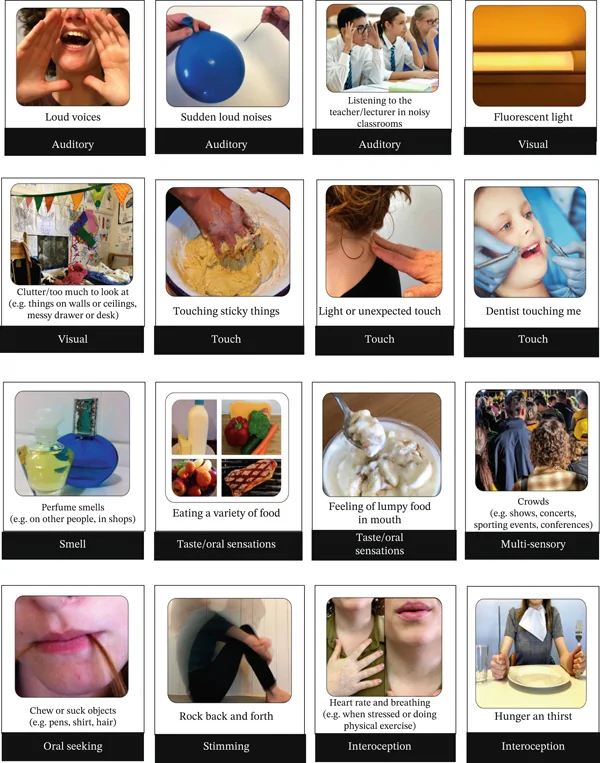
Example MYSET cards∗. ∗Original photographs or photographs accessed through a Creative Commons License with the written permission of the licensor.

Consistent with the semi‐structured interview approach suggested by DuBois et al. [8], MYSET was designed to facilitate semi‐structured conversations [18] based on the sorting of cards with photographs (see example photographs in Figure 1). As MYSET yields detailed qualitative information rather than quantitative scores, it aims to augment rather than replace standardised quantitative sensory processing measures, which may be required for diagnostic purposes [3].

The first version of the tool was a semi‐structured interview augmented by visual cues used to gather data for a qualitative study exploring the sensory experiences of autistic adolescents [19]. This version was designed to facilitate discussion around sensory experiences, with pictures and prompt questions in each sensory domain. Many occupational therapists working within a service provider for autistic people, wanted to use the tool clinically. It was therefore further developed into a booklet form called the Sensory Experiences Interview that was used regularly within the organisation over many years and was iteratively revised many times in response to feedback from autistic children and their family members, autistic adults and therapists.

To further enhance its usability, the Sensory Experiences Interview was re‐developed into a card‐sort tool and renamed My Sensory Experiences Tool (MYSET). The picture‐based card‐sort methodology was chosen because it has been successfully used in numerous other tools for children and adults including some autistic individuals, to measure self‐reported participation and competence in everyday activities and to facilitate goal setting. For example, other card‐sort tools include the Perceived Efficacy and Goal Setting System [20] for children aged 5–9 years, the Pediatric Activity Card Sort [21] for children aged 6–12 years, the Adolescent and Young Adult Activity Card Sort [22] for young autistic people, and the Adolescent Adult Goal Setting Tool [23] for autistic adolescents and adults including people with mild intellectual disability. Card‐sorting enables participants to focus on one item at a time, which is advantageous for autistic people whose cognitive style is often characterised by mono‐tropism (hyperfocus on a singular interest at any given time) [24]. Cards can also be readily sorted in order of priority with the aim of identifying the sensory experiences that have the largest impact on their lives. The service provider funded the development and evaluation of the tool, with all profits from the sale of the tool being reinvested into services for autistic children and adults, and further autism research.

### 2.2. Considerations that Guided the Development of MYSET

#### 2.2.1. Consideration 1: Alignment With a Neurodiversity‐Affirming Paradigm

A neurodiversity‐affirming paradigm views neurological differences between people as different expressions of human behaviour and functioning [25]. Sensory differences are therefore perceived to be part of the person’s identity. In the words of Dunn [26], “*Sensory processing patterns are reflections of who we are. These patterns are not a pathology that needs fixing*.” Rather than perceiving deficits to be a fault that resides within the individual, a neurodiversity‐affirming paradigm recognises that challenges may be the consequence of a mismatch between the person and their environment [25]. As a neurodiversity‐affirming approach positions the autistic person as the ‘expert’, the person’s voice in identifying what they consider important for a meaningful life is central to the MYSET process.

#### 2.2.2. Consideration 2: Accessibility to a Diversity of Autistic People

The goal was to design a tool that is accessible to autistic people with a range of ages, abilities, and communication skills. Courchesne et al. [27] found that pictures enhanced the capacity of autistic adolescents to respond to interview questions. In the latter study, both the autistic participants with low non‐verbal intelligence and/or minimal verbal language, and the autistic participants with average to high non‐verbal intelligence benefitted from the use of pictures. Pictures were therefore incorporated throughout the tool with the aim of making it accessible to autistic people with both low and high levels of intelligence. As evidence suggests that children as young as 5–7 years can reliably self‐report their experiences [28], we anticipated that the tool would be accessible to children in this age group. We also predicted that some people with intellectual disability would be able to use the tool independently. We considered it important to presume competence by giving the person the opportunity to use the tool independently [29]. Consequently, all participants including young children and people with intellectual disability were encouraged to sort the cards by themselves in the first instance. Nevertheless, as very young children and some people with high support needs may be unable to use the tool independently, we developed proxy‐report options to be completed by caregivers or educators.

As outlined in the MYSET User Manual, the tool can be used flexibly to accommodate people with limited attention spans. For example, the tool may be administered over several sessions by focusing on one or more sensory domains at a time. Another option is to first ask a family member to complete the caregiver/educator version to ascertain the sensory experiences most likely to be impactful, and then to use this information to select a smaller subset of cards to be sorted by the autistic person.

#### 2.2.3. Consideration 3: Selection of Items Based on First‐Hand Accounts

The tool was designed to comprehensively cover sensory experiences that have been reported to impact the everyday lives of autistic people. The selection of items in MYSET was underpinned by first hand accounts sourced through a review of 33 qualitative publications and five autobiographies of autistic people. Illustrative quotes of autistic people, their caregivers or teachers drawn from these publications, are shown in Supporting Information S1 (see link to the supporting information section at the end of this article). We organised the quotes into categories in each sensory modality representing (a) hypersensitivities, (b) preferences, (c) sensory interests, (d) filtering challenges, (e) sensory seeking and self‐stimulatory behaviours (stimming) and (f) reduced or heightened awareness. For example, an autistic participant described his hypersensitivity to bright artificial lighting: “*My eyes have struggled in fluorescent light or unnatural light … that sort of stimulus becomes painful after a while*.” ([30], p. 575). This and other similar quotes led to the development of cards that illustrate bright lights including the following: ‘*Fluorescent lights*’ and ‘*Lighting in classrooms or workplaces*’. Others described sensory seeking or stimming behaviours [31, 32]. For example, an autistic participant in a study by Kapp et al. [33] described her craving of vestibular input: “*I remember as a child spinning all the time and loving spinning and loving swinging … I also realised that there was a point where it wasn’t acceptable to be spinning anymore … so it actually still feels glorious if there’s nobody around and … I can spin*.” We therefore developed cards that illustrate seeking of vestibular input including ‘*Swing (e.g. on a swing or hammock)*’ and ‘*Spinning (e.g., on an office chair)*’.

### 2.3. Description of MYSET

The goals of MYSET are the following:

• facilitate conversations with autistic people about their sensory experiences.
• enable others in their lives (e.g., family members, teachers and employers) to understand their sensory experiences.
• facilitate collaborations between autistic people, family members and professional practitioners about accommodations that are compatible with the autistic person’s lifestyle.

Figure 1 shows examples of the MYSET cards, which represent sensations that commonly occur in everyday life, including some that occur across multiple environments (e.g., loud voices) as well as specific environments (e.g., dental surgeries). Both original photographs and photographs accessed through a Creative Commons License with the written permission from the licensor were used to illustrate the sensory experiences depicted in the cards.

**Figure 2:**
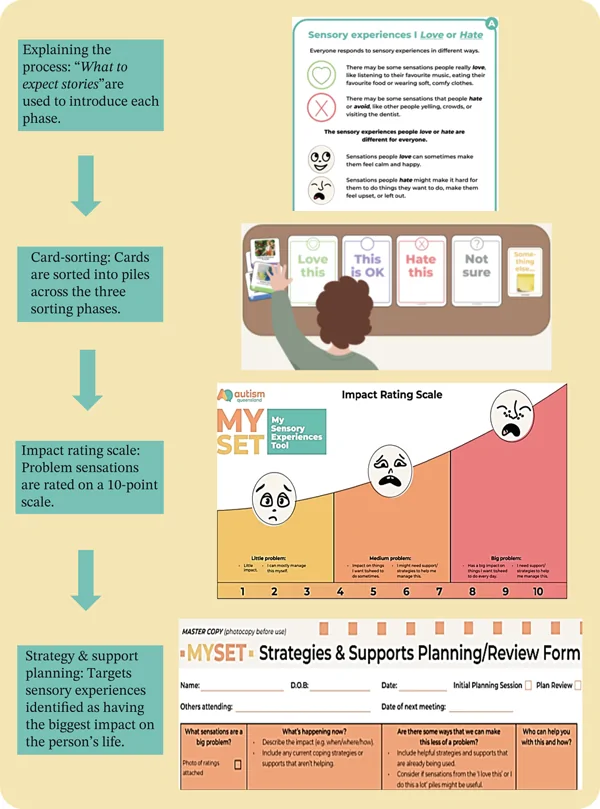
Four steps in the MYSET process.

Figure 2 shows the four steps in the MYSET process. First, MYSET is introduced to the participants using ‘What‐to‐Expect Stories’, which are easy‐read stories with pictures that explain what is meant by sensory experiences, how the tool is used, how it can help and what to expect in each stage of the process.

**Figure 3:**
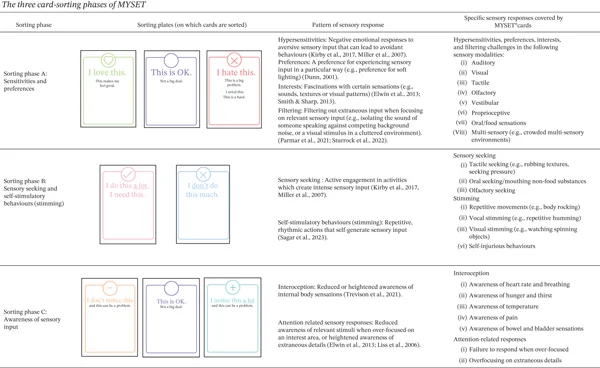
The three card‐sorting phases of MYSET.

The second step in the MYSET process is card sorting, which is completed in three phases, each with a different set of baseplates onto which the cards are sorted (see details in Figure 3). The baseplates correspond to the nature of the sensory experience being explored. The cards enable the participant to choose the direction of their response thereby accommodating heterogeneity. For example, some autistic people enjoy smelling perfumes, while others find perfumes offensive.

In Phase A, 60 cards representing hypersensitivities, preferences, interests and filtering challenges are sorted onto the following three sorting plates with the simple wording: (a) “*I love this. This makes me feel good*.”; (b) “*This is ok. Not a big deal.*” or (c) “*I hate this. This is a big problem. I avoid this. This is hard*.” Hypersensitivity is described as a negative emotional response or avoidance of sensory input that the person finds aversive [31, 32]. Sensory preferences describe the way a person typically prefers to experience sensory input (e.g., preferring mood lighting) [26], while sensory interests describe fascinations with particular types of sensory input (e.g., specific sounds, visual patterns, or smells) [34, 35]. Filtering challenges refer to difficulties filtering out extraneous input in order to focus on relevant sensory input, such as focusing on the sound of someone speaking against competing background noise [36, 37] or focusing on important visual information in a visually cluttered environment [38, 39].

In Phase B, 15 cards representing sensory‐seeking and stimming behaviours are sorted onto the following two sorting plates with the simple wording: (a) “*I don’t do this much*.” and (b) “*I do this a lot. I need this*.” Sensory seeking refers to an unusual craving or engagement in activities, which create intense sensory input [31, 32]. ‘Stimming’ refers to repetitive, rhythmic actions that self‐generate sensory input such as repetitive movements (e.g., hand flapping) or vocal stimming (e.g., repetitive humming) ([33, 40]. Sensory seeking and stimming are related concepts, although stimming is characterised by more repetition and rhythm. Stimming behaviours are perceived by autistic people to help with self‐regulation when they experience sensory overload, dysregulated thoughts or uncontainable emotions [33].

In Phase C, eight cards are sorted onto the following three sorting plates with the simple wording: (a) “*I don’t notice this, and this can be a problem.*”, (b) “*This is OK. Not a big deal*” and (c) “*I notice this a lot, and this can be a problem.*” The Phase C cards represent reduced or heightened awareness of internal bodily signals or interoception (i.e., heartbeat, pain, hunger, thirst, temperature or bowel and bladder sensations) [41]. Attention‐related sensory responses as described by Elwin et al., [34] and Liss et al. [42] are also explored including reduced awareness of important input (e.g., someone talking to them) when over‐focusing on an area of intense interest, or heightened awareness of extraneous details such as the hum of refrigerator. Each sorting phase includes a ‘*Something else*’ card, which allows the participant to identify any additional sensations not already captured in the cards, and an “*I’m not sure—it depends*” card, which allows the participant to describe contextual factors influencing their sensory response.

The third step is to prioritise the sensory experiences identified as problematic during the card sort, in terms of the degree of impact, using the 10‐point Impact Rating Scale. The participant is asked to rate the impact, taking into consideration the extent to which it impacts on things they need or want to do in their daily life, and whether they have management strategies that work for them.

The fourth step involves the use of the Strategy and Support Planning process to collaboratively design accommodations to minimise the impact of sensory experiences identified as having the biggest impact on their life. Positive sensory experiences that the person likes or seeks out may be suggested as a means of coping with unpleasant sensory experiences (e.g., eating a favourite food to help cope with a stressful experience such as a haircut). The Strategy and Support Planning process forms the basis of a MYSET report (example reports are provided in the MYSET User Manual).

Professional practitioners play a key role in facilitating the conversation by answering questions and clarifying what the person means. For example, if the person places a card on the “*I’m not sure. It depends*” baseplate, the professional practitioner seeks clarification. The MYSET User Manual prompts professional practitioners to be mindful of trauma‐informed practice by considering instances in which the MYSET process triggers memories, such as shame and punishment associated with stimming. The professional practitioner therefore needs to explore the person’s sensory experiences in supportive ways (e.g., whether they feel comfortable stimming in the presence of others or whether they feel the need to suppress these behaviours).

MYSET can be used by a range of professional practitioners (e.g., occupational therapists, teachers or case managers). The MYSET User Manual recommends that ideally, if professional practitioners have access to an occupational therapist, they should seek the occupational therapist’s advice on strategy planning. Nonetheless, the need for some professional practitioners to use the tool in situations where access to an occupational therapist is unavailable, is acknowledged (e.g., teachers working in mainstream schools). The MYSET User Manual and ‘What to Expect Stories’ explain the process very clearly. The tool can therefore be used without training. An instructional video is, however, currently being developed for professional practitioners seeking additional information on the use of the tool.

As we considered it important to determine whether MYSET was perceived to be beneficial in supporting the sensory challenges and preferences of autistic children and adults, a qualitative study exploring the perspectives of autistic people, their family members and professional practitioners of the usefulness of the tool was conducted.

### 2.4. Research Aim

The study aimed to explore the perspectives of autistic people, family members and professional practitioners on the usefulness of MYSET and to explore ways that it could be improved. The research questions were as follows:

• How do autistic people, family members and professional practitioners perceive the usefulness of the information gained using MYSET?
• How do autistic people, family members and professional practitioners perceive the utility of MYSET processes, including the what‐to‐expect stories, the card‐sorting, the Impact Rating Scale and the Strategy and Support Planning Process?
• Do autistic people, family members and professional practitioners perceive MYSET to be helpful in enabling others in the person’s life to understand their sensory experiences?
• Do autistic people, family members and professional practitioners perceive MYSET to be helpful in enabling the generation of strategies and accommodations that are compatible with the autistic person’s lifestyle?

### 2.5. Ethical Considerations

Ethics aproval was obtained from the University of Queensland Human Research Ethics Committee (2009001253; 2022/HE000535). All autistic adults, professional practitioners and parents of autistic children provided informed written consent, while autistic children provided written assent. Autistic participants received an ‘Easy English’ version of the participant information sheet with picture cues.

### 2.6. Methodology

A qualitative descriptive approach was used within a constructivist paradigm emphasising the participants’ subjective experiences [43]. Consistent with participatory research practice guidelines [44], the research team included two autistic investigators who advised on the accessibility of the participant information and consent forms, interview questions and interview procedures. This project complied with both the Standards for Reporting Qualitative Research (SRQR) [45] and the Consolidated Criteria for Reporting Qualitative Research guidelines (COREQ) [46]. Supporting Information S2 contains completed SRQR and COREQ checklists (see link to the supporting information section at the end of this article).

#### 2.6.1. Participants

Eighteen professional practitioners, five autistic individuals and four family members were recruited through the networks of Autism Queensland a service provider for autistic people, according to the following criteria: (a) professional practitioners who had facilitated the use of MYSET, (b) autistic participants who had used MYSET and (c) family members who had supported their child in using MYSET. All participants provided information on their age, gender, highest educational level, geographical location and language spoken at home. Professional practitioners provided information on their work context and professional experience. Autistic participants also provided information on their co‐occurring conditions and the professionals who had diagnosed them with autism spectrum disorder.

The professional practitioners included 15 with an occupational therapy background and three with an educator background (see Table 1). The professional practitioners who were Autism Queensland employees were purposively sampled to include practitioners working in a range of service areas, including individual therapy, positive behaviour support, autism‐specific schools, mainstream school consultancy, mental health services, supported accommodation and assessment and diagnostic services. We also aimed to recruit professional practitioners seeing clients with a range of cognitive/communication abilities. The 18 professional practitioners had used MYSET with 41 autistic clients aged 5–49 years, including five with a suspected cognitive/language impairment, and seven with a diagnosed intellectual disability.

**Table 1:** Demographics of professional practitioners.

Demographic information		*n* (%) (*n* = 18)
Age	23–30 years	12 (66.7%)
	30–40 years	4 (22.2%)
	40–50 years	1 (5.6%)
	50–60 years	1 (5.6%)

Gender	Female	18 (100%)
	Male	0 (0%)
	Non‐binary	0 (0%)

Professional role	Occupational therapist	14 (77.8%)
	Occupational therapy student	1 (5.6%)
	Developmental educator	1 (5.6%)
	Behaviour support practitioner and educator	1 (5.6%)
	Inclusive education consultant (educator)	1 (5.6%)

Highest level of education	Currently completing Bachelor degree	1 (5.6%)
	Bachelor degree	12 (66.7%)
	Master’s degree	5 (27.8%)

Geographical location	Australian capital city	11 (61.1%)
	Regional city	7 (38.9%)

Years of experience	0 (occupational therapy student)	1 (5.6%)
	1–4 years	10 (55.5%)
	5–10 years	4 (22.2%)
	10–15 years	2 (11.1%)
	16–20 years	2 (11.1%)

Work context	Individual therapy team	10 (55.5%)
	Positive behaviour support team	2 (11.1%)
	Autism‐specific school	2 (11.1%)
	Mainstream school consultancy team	1 (5.6%)
	Mental health support team	1 (5.6%)
	Supported accommodation team	1 (5.6%)
	Assessment and diagnostic team	1 (5.6%)

Experience in using sensory processing assessments	Sensory Profile suite	9 (50%)
	Sensory Processing Measure suite	0 (0%)
	No previous use of sensory processing assessments	5 (27.8%)
	Missing	4 (22.2%)

Level of experience using sensory processing assessments with individuals with autism or developmental disabilities	Not at all experienced	1 (5.6%)
	A little bit experienced	4 (22.2%)
	Somewhat experienced	4 (22.2%)
	Quite experienced	5 (27.8%)
	Missing	4 (22.2%)

Cognitive/language capacity of clients who used MYSET (*n* = 41)	Children (aged 5–17 years)	29 (71%)
	Adults (aged 18–49 years)	12 (29%)

Cognitive/language capacity of clients who used MYSET (*n* = 41)	No intellectual disability or language disability	29 (71%)
	Suspected cognitive or language impairment	5 (12%)
	Diagnosed intellectual disability	7 (17%)

The autistic participants were purposively sampled to include a range of ages (10–54 years) (see Table 2). With the aim of supporting transparency and the interpretation of the participant’s quotes [45, 46], the characteristics of autistic participants likely to impact on their functional capacity were assessed. The assessments included the Social Responsiveness Scale (2nd Edition) (SRS‐2) [47], which estimates the level of autistic traits and the Adaptive Behaviour Assessment Scale (3rd edition) (ABAS‐3) [48], which measures adaptive skills. The intellectual ability of three autistic participants was estimated using the Kaufman Brief Intelligence Test (2nd edition) (K‐BIT‐2) [49]. The intellectual ability of two participants was not formally measured but was assumed to be above average due to their high level of academic achievement (one with a master’s degree and one with a PhD). Ideally, we would have liked to interview more autistic children and autistic adults with intellectual disability who had used the tool, but we found that these participants preferred parents or professional practitioners to report on their behalf.

**Table 2:** Demographics of autistic participants.

Participant number	Age	Gender	Professional who diagnosed them with autism spectrum disorder	Highest level of education	Type of school attended	Geographical location	Language spoken at home	Co‐occurring conditions			Level of social impairment measured by social responsiveness scale (2nd ed.)	Level of adaptive skills measured by adaptive behaviour assessment system (3rd ed.)	Estimated IQ measured by Kaufman brief intelligence test (2nd ed.)
								ADHD	Anxiety disorder	Depression			
AP01	34	Non‐binary	Psychiatrist	Master’s degree	Regular mainstream school	Australian capital city	English	✓	✓	✓	Severe range	Below average	^a^
AP02	10	Male	Psychologist	Still in primary school	Regular mainstream school	Australian capital city	English	✓			Severe range	Low	IQ composite score 124 (above average range)
A03	54	Non‐binary	Psychiatrist	Tertiary education diploma or degree	Regular mainstream school	Australian capital city	English	✓	✓	✓	Moderate range	Below average	IQ composite score 116 (above average range)
AP04	22	Female	Psychiatrist	Tertiary education diploma or degree	Regular mainstream school	Within 2 h of a regional or capital city	English	✓	✓	✓	Severe range	Low	IQ composite score 106 (average range)
AP 05	31	Female	Psychologist	PhD	Regular mainstream school	Regional city	English				Within normal limits	Below average	^a^

^a^Not formally assessed but assumed to be above average due to high level of academic achievement.

The four family member participants (three mothers and one father) were purposively sampled to include parents of children with a diversity of communication abilities, ranging from age‐appropriate communication skills to minimally speaking. They were aged 41–45 years, were all tertiary educated and spoke English at home.

#### 2.6.2. Fidelity of Use of MYSET

The research team conducted fidelity observations of the MYSET sessions of the autistic participants, using a fidelity checklist detailed in Supporting Information S3 (see link to the supporting information section at the end of this article). Observers were prompted by the checklist to note whether the participants (a) were encouraged to ask questions, (b) had led the card sort and (c) were encouraged to discuss the impact of their sensory experiences using the Impact Rating Scale. They also observed the use of clear concrete language and the use of the participants’ own words on the Strategy and Support Planning and Review sheets. All MYSET sessions met the fidelity requirements.

#### 2.6.3. Data Collection

Qualitative data was gathered through semi‐structured interviews with the autistic participants and family members, and focus groups with the professional practitioners, conducted via Microsoft Teams videoconferencing (version 25122.1415.3698.6812). The reasons for using focus groups with the professional practitioners were firstly to allow time‐efficient gathering of information from multiple participants, and secondly to explicitly use group interactions as part of the data gathering process [50]. Most of the professional practitioners had used MYSET with multiple clients with a range of ages and co‐occurring conditions in a variety of practice contexts (e.g., school or individual therapy). Group interactions within the focus groups enabled them to draw inferences about the tool’s applicability to diverse practice contexts. There were five professional practitioner focus groups that were approximately 1‐h in duration and that included between two and six participants. Interviews were used with autistic participants and family members as they allowed each participant to discuss their unique individual experiences in detail, although they could not comment on the tool’s applicability to people of different ages or experiences. The interviews ranged in duration from 25 to 70 min (autistic participants) and 30 to 70 min (family members). The interview and focus group questions, which are provided in Supporting Information S4 (see link to the supporting information section at the end of this article). Strategies to maximise the autistic participants’ engagement in interviews included the use of vocabulary to match their communication style (e.g., the interview questions were rephrased for the child participant by using shorter sentences and simpler wording). All participants were introduced to each phase of the MYSET process through the ‘What to expect stories’ and were made aware of the ‘*Stop*’ and ‘*Take a break*’ cards that they could use if feeling overwhelmed [51]. If the participants were interviewed online, they were verbally reminded that they could withdraw from the interview, stop or take a break at any time. The interviews and focus groups were conducted by JA, VT and CD, who had between 4‐ and 18‐years’ experience in autism research, in addition to being allied health professionals with extensive autism‐specific experience. Further details on the background of the researchers are available in Supporting Information S2.

#### 2.6.4. Researcher Positionality

JA, VT, JH and JC all have an occupational therapy background, which shaped the daily life participation focus of MYSET [52]. JA and VT occupied an insider position, as they were responsible for the development of MYSET, giving insight into the rationale behind aspects of the MYSET design. The risk of bias was mitigated by engaging researchers from a local university, JH and JC, who provided an outsider perspective by reviewing and questioning the interpretation of the data through processes such as double coding. CD is an audiologist with specialist knowledge of the auditory processing challenges of autistic people. ER and NB were research officers with bachelor degrees majoring in psychology.

#### 2.6.5. Data Analysis

The written transcripts generated by the video‐conferencing platform were reviewed for accuracy and anonymised before analysis. Data was analysed using inductive content analysis [53]. Inductive content analysis was considered appropriate because this study aimed to explore specific and practical perspectives on using the tool in clinical practice without imposing predefined categories [54]. All transcripts were read thoroughly to enhance familiarity with the data. JA manually coded the data into subcategories and categories, and developed a preliminary coding dictionary with clear descriptions of each code. JH, who provided an outsider perspective, independently re‐coded 100% of the data. This involved re‐assigning codes to the data without access to JA’s coding decisions, using a consensus coding method. JA and JH met to review, discuss and collaboratively refine the codes [55]. The codes were clarified or redefined, until agreement on the final codes was reached. For example, the data on the limitations of the tool and suggested improvements were recoded into separate categories, allowing the researchers to clearly identify ways to improve the tool. As no new categories or sub‐categories emerged in the later interviews and focus groups, we judged that the point of data saturation had been reached. Strategies to improve rigour included the following: (a) triangulation by gaining the perspectives of multiple stakeholder groups, (b) having two coders independently review the findings and (c) a member checking procedure which involved one autistic person (AP01), one family member (FM04) and one professional practitioner (PP08) reviewing the synthesised and analysed data. The latter participants were selected as member checkers, as they had been involved in the most recent interviews or focus groups and were thus the most likely to recall the details of the discussion. The member checkers agreed that the way that their comments had been coded accurately represented their experiences.

## 3. Findings

**Figure 4:**
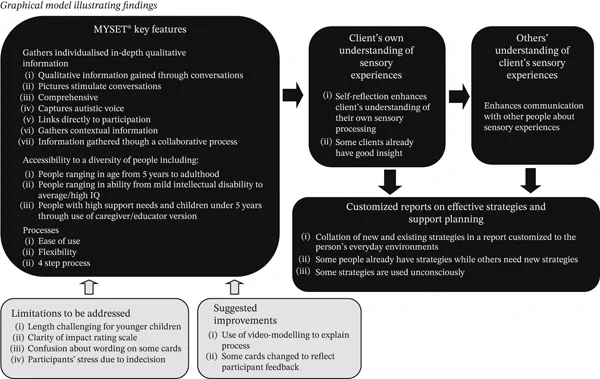
Graphical model illustrating findings.

The findings are graphically illustrated in Figure 4 (six categories with three sub‐categories under Category 1). The information in the black squares in Figure 4 shows the key features and outcomes of MYSET, while the information in the grey squares shows participant perceptions of limitations and suggested improvements. Participants were assigned identifier numbers corresponding to their participant group (autistic participants = AP, family member = FM, professional practitioner = PP). Participant quotes are used to illustrate each subcategory.

### 3.1. Category 1: MYSET Key Features

#### 3.1.1. Sub‐Category (1): Gathers Individualised In‐Depth Qualitative Information

Professional practitioners appreciated the depth of qualitative information gained through conversation, including PP08: “*I think that’s a real benefit of the tool and that kind of qualitative information we get through the process*. *There was lots of conversation, which was really valuable. I previously haven’t had that using other sensory assessments.”* PP14 described the way that MYSET had supported her client to share a more detailed perspective of his sensory experiences:

*He’s the sort of client where you might ask a question, and he might say “Ohh maybe” or “I don’t know”. But if he’s sorting them, he’s able to say yes or no. We’ve had some really interesting conversations and even his mum has been like, “Wow, I never knew that that was what was going on for you.”*

Some professional practitioners attributed the capacity of the tool to generate rich qualitative data to the effectiveness of the pictures in facilitating conversations. PP15 reported that her client said:

*This is so good; you should do this with everyone. Having the cards with all of the pictures makes it so much easier to be able to explain what is going on for me, rather than having to come up with it myself.*

AP03 explained that *“the pictures helped with the memory of incidents.”* Participants perceived that MYSET generated comprehensive information. FM01 said “*It was very comprehensive … I didn’t feel like anything was missing … if anything, there were … things that I hadn’t thought of.”* AP05 remarked that the time taken to use the tool was necessary to achieve comprehensiveness:

*I think that patience needs to be exercised, because you are trying to understand and discern a person’s experience and how they see the world. It’s gonna take as long as it’s gonna take, and that’s OK because … it needs to be comprehensive*.

Participants also commented on the capacity of the tool to capture the voice of autistic people, including PP15: “*What I was able to do was identify student voice very, very clearly…*. *these students were able to very clearly ask more questions about why some of these things that they had identified might have been affecting them*.” FM01 highlighted the importance of the autistic voice: “*Their voice is what’s important. …. The sensory experiences where autistic people are totally misunderstood. …Yet, it’s so important. Like it’s just such a game changer.*” Some family members liked the way that the MYSET results could be linked directly to daily life participation. FM03 compared it to a previous experience with a standardised sensory processing questionnaire, which resulted in:

A *label that was given – you know, sensory seeking.* …*This [MYSET] is much more valuable. The conversations that would come from unpacking those things that are having an impact and thinking about adjustments or strategies that we can use*.

PP01 commented on the challenges that professional practitioners experience in relating standardised sensory processing questionnaire results to daily life participation: *“The Sensory Profile where you gotta go and try and decipher what the results are telling you. Whereas this one [MYSET] is quite clear*.” Some participants highlighted the importance of discussing the context of the sensory experience, as participants often say: “*It depends…*” AP04 remarked: “*I would just explain to her—how I am putting it in this group because I feel like probably it’s most of the time in this group, but it can change depending on the situation.*” Some autistic participants described an appreciation of the collaborative process with a professional facilitator, including AP05: “*The difference in this tool …is that we have two people that are involved actively in the process, so they’re co-creating something together, which I think is great. It’s like a therapeutic process in that way.*” Some appreciated the presence of a professional practitioner to clarify areas of uncertainty including AP03*: “A good contrast* (compared to standardised sensory processing questionnaires) *is you have a clarification of making sure what you meant was what you meant.*” Most participants said that having a professional practitioner made the process easier, including AP01: “*I think it’s better having someone else there, in particular, a person who’s trained in that sort of area …there are a lot of complexities.”*

#### 3.1.2. Sub‐Category (2): Accessibility to a Diversity of Clients

Some professional participants expressed surprise about the capacity of young children and clients with mild intellectual disability to use the tool. PP04 remarked:

*It was nice with my client. He has some level of expressive language difficulties and some difficulties with kind of expressing his emotions. He can have limited focus and when he decides that he’s done with an activity, he moves on pretty quickly. But he engaged with the card sort for 50 minutes …which surprised me…. He’s only five.*

PP06 said *“The client that I did it on with the intellectual disability, he actually attended quite well to it.”* Autistic participants who were tertiary educated and highly articulate, were also very engaged by the picture‐based card sorting, including AP05: “*Yeah, like looking at an image. ‘Ohh, I had an experience like that.’ It’s just like look at something— there’s a thought that comes up. Not really the words …the imagery is more powerful.*” There were two cases, in which proxy reporting options were needed for non‐speaking clients. First, a team of support workers who cared for a gentleman in a supported accommodation facility, worked together to complete MYSET:

*The gentleman is currently non-speaking, and he does not have any substantive functional communication system …and also can find it quite difficult to attend to visuals. … We (the support team) used the card sort to get an understanding of the things that he found particularly aversive, or wasn’t particularly interested in …My overall impression of the tool was that it was an amazing way to collaboratively come together as a support team who knows this gentleman really well …It provided everybody with an opportunity to give input and generate a list of recommendations for different things that we could try.*

Second, supports for an 8‐year‐old non‐speaking child were based on two sources of information. The child sorted the cards himself but as he was unable to articulate his reasons, his mother also completed the caregiver version of MYSET. His mother commented on her son’s card‐sorting:

“*Oh wow!’ I didn’t realise that he would be able to do that. I was very keen for him to have a go because (Child)′s minimally verbal, so it’s hard to know what he knows.*” She also commented on her own experience:

*As a parent of a minimally verbal child, I’m making a lot of assumptions which I’m very aware of, so while there’s some things that are really clear, like, I know that he hates having his haircut … other things are not as obvious…I was thoughtful in my responses … I even put a ‘Not sure’ in the middle*.

#### 3.1.3. Sub‐Category (3): Processes

Participants from all three groups described the tool as easy to use, including PP10: “*It’s quite easy to follow with the manual having pictures and photos.*” PP01 said “*The Sensory Profile is very confusing, those five options [Likert scale] confuse people. My Sensory Experiences is a lot better - takes out the jargon.*” Professional practitioners valued the flexibility of the tool in that they could “*do it in the way that works for each individual client. Being able to pick the cards that you’re actually targeting in that moment*”. The four‐step process was perceived to be useful, including the introduction to MYSET using the What‐to‐Expect Stories, as articulated by PP01: “*The example script provided is really useful… The person I completed it with understood why we were doing it*”. Autistic participants appreciated having both the pictures and the wording on the cards. AP04 said “*They (the cards) were quite easy to understand …having a picture with writing can help me understand the meaning behind the words.*” The Impact Rating Scale was also perceived to be worthwhile. FM03 said *“Allowing him to come up with a ranking system so he can explain what impacts him the most is a big, big benefit.”* The Strategy Planning and Support process was also perceived to be an important part of the process. FM01 said that the Strategy Planning and Support process “*gave us a structure to work through.*”

### 3.2. Category 2: Client’s Own Understanding of Sensory Experiences

The autistic participants talked about the way that MYSET had prompted them to reflect on their own sensory experiences. For example, AP04 said:

*I was able to learn a bit more about myself … I did not really think about how certain things affected me before and then that made me think about it. I did not…realize [my sensory experiences] were affecting me as much as they were. I was able to talk about that with my partner and my friends, which was good.*

Some autistic participants who had done a lot of self‐reflecting explained that they already had good insight into their sensory responses. AP01 said “*I think it would be much more helpful for people who didn’t have as much insight as I’ve done a lot of time self-reflecting.*” However, these participants valued the way that MYSET helped them talk to others about their sensory experiences (see Category 3 below).

### 3.3. Category 3: Others’ Understanding of Clients’ Sensory Experiences

Some participants talked about the way that MYSET had enabled the autistic person to help others understand their sensory preferences and needs. AP01 commented:

“*Having a concise way to talk to someone else about (my sensory experiences) could help alleviate some of those sort of barriers … to figure out how to explain*.” FM04 said:

*We both (parents) found it quite insightful. Really just to be able to communicate at that level by pointing to the graphics … this tool enabled him to open up … I found it quite fascinating. It’s made us more aware of certain things …. certain noises can have an impact and result in certain behaviours.*

Participants talked about how other people’s awareness of their sensory experiences improved their capacity to access many areas of daily life. AP01 said:

*[Others’ understanding] is quite important for me ‘cause a lot of my ability to interact in the world is based around whether or not I’m able to cope with the sensory experiences - so I think people knowing about sensory sensitivities … can be so beneficial for being able to access just everything really.*

FM03 learned for the first time that sensory experiences were less aversive if his son was in control of the sensation:

*That was a bit of a light bulb moment. …if you can get him to do the vacuuming, he does not mind it. … And so maybe he can use that to help him going forwards, that if he …can better control or regulate what’s going on, then he feels he’s in control and that helps minimize the impact on him.*

### 3.4. Category 4: Customised Reports on Effective Strategies and Support Planning

The strategies and support planning were collated in a report that was customised to the participants’ everyday environments such as their home, workplace or school. FM01 appreciated the development of effective strategies for school: *“The strategies that she (therapist) has given us are excellent. … I think those strategies are actually all achievable as well for the school.”* FM04 also considered this heightened awareness to be important for her child’s participation at school: *“Having reviewed the (MYSET) report … gave me further insight into some of the things that do set him off at school. It was thought provoking. So, the report from this tool fits nicely with the (school) advisory report meeting.”* AP04 appreciated receiving the MYSET report that detailed a range of beneficial strategies: *“I really enjoyed getting the actual stuff back afterwards and then seeing what had been written on the strategies we talked about that would help me.”* Some autistic participants became aware of strategies that they were already using unconsciously, including AP05: “*It brought to light my strategies because a lot of the things that I do are just unconscious.”* Others reflected on both the strategies that they already use, as well as the need to develop new strategies, including AP03*: “I may automatically have some strategies accumulated over the years that sort of lessen the blow. And then there’s probably other areas that I still need strategies in.”*

### 3.5. Category 5: Limitations To Be Addressed

Although the feedback was generally positive, some participants mentioned limitations that warrant further consideration. First, the tool was perceived to be excessively long for younger children. PP13 said “*Definitely with the youngest students - I think you have to split it over a couple of sessions because he just couldn’t maintain attention for that long*.” One autistic participant was uncertain about how to rate the impact of the sensory experience:

*I was struggling to differentiate between the functional impact, the emotional impact and the management strategies…. Take fireworks for example, the emotional impact is significant for me, but the functional impact probably would not be as much, because I do not really go to fireworks that often. But I would like to go with my mother. The strategies I have is essentially just avoiding it.*

Some parents suggested that the wording on a few cards was too challenging for younger children. FM01 said: *“(the card about) background noise… it was quite a lot of words.*” A few clients were stressed by difficulties in making decisions about how to sort the cards as indicated by parent FM02: “*There were too many cards to sort for someone that finds it difficult to make choices or worries about getting it wrong…although (the professional practitioner) said over and over again, there’s no right or wrong answer.”*

### 3.6. Category 6: Suggested Improvements

Participants suggested two ways in which the tool could potentially be enhanced. First, two participants recommended sending a video of the tool being used to autistic participants in advance, as articulated by FM01*: “Video modelling is what you need. He could have access to that to watch however many times he wanted prior and then on the actual day.”* Second, some autistic participants suggested additional cards, such as escalators, warning beeps on vehicles and driveway closures, light touch and additional cards on clothing (*seams inside socks)* and fabric *(ribbed fabric such as corduroy).*

## 4. Discussion

The findings show that the nature of information gained through MYSET differs markedly from the information typically gathered through standardised sensory processing questionnaires. The professional practitioners perceived that the semi‐structured interview format augmented by visual cues as recommended by Dubois et al. [8] yielded more nuanced, qualitative narratives, grounded in the person’s daily life experiences. The practitioners reflected that the tool had enabled them to capture the autistic voice, positioning the autistic person as the expert, thereby aligning with neurodiversity affirming principles [25]. This contrasts with the data gathered through standardised assessments, where the autistic person’s responses are confined to items in ‘tick and flick’ questionnaires. The professional practitioners also observed that currently available self‐report sensory questionnaires can be confusing for some clients to complete, particularly if they experience communication differences [11, 12].

The conversational nature of the tool allows the autistic person to expound on their personal experiences of their sensory world, providing direct links to their unique daily life experiences. MYSET gave the professional practitioners the opportunity to explore activities and environments that are meaningful to the individual (e.g., their sensory experiences at school or in the workplace), thus recognising that challenges may be the consequence of a mismatch between the person and their environment [25]. As observed by Lucas et al. [17], clinicians often struggle to link quantitative sensory processing scores with a plan to support the person’s daily routines. Practitioners therefore find it necessary to supplement the information gathered through standardised assessments with additional parent and/or teacher interviews, and observations [5]. The voice of the autistic person is, however, often missing from this process, either because children’s sensory processing assessments are completed by caregivers, or because self‐report sensory checklists are inaccessible to many adults due to the language demands. Standardised sensory processing assessments are essential for diagnostic purposes but may be insufficient when used on their own for the design of supports to overcome everyday sensory challenges. MYSET bridges this gap by gathering individualised in‐depth qualitative information on everyday sensory experiences directly from the autistic child or adult.

As noted by MacLennan et al. [1], autistic participants reflected that their sensory responses vary considerably depending on contextual factors. The “*I’m not sure—it depends*” card was used frequently, as many autistic participants considered it essential to explain the contextual factors impacting their experiences. Others appreciated the collaborative process with a professional practitioner that provided opportunities to clarify uncertainties, and to explain the idiosyncrasies of their sensory experiences.

Autistic participants highlighted the importance of others in their life understanding their sensory experiences. This also corresponds with the findings of McLennan et al. [1] who reported that other people’s misunderstanding of sensory experiences can have a negative impact on autistic people, whereas close relationships with others who understand their sensory experiences can be a source of support. The MYSET Strategy and Support Planning process provides an opportunity for autistic participants and professional practitioners to collaborate on ‘finding the words’ to explain their sensory experiences to others. MYSET reports can be tailored to specific environments such as school, workplace or home by detailing the most impactful sensory experiences within these environments and recommending context‐specific strategies.

MYSET was perceived to be accessible to a diversity of people including people ranging in age from 5 years to adulthood and people with abilities ranging from mild intellectual disability to average/high IQ. Consistent with the findings of Courchesne et al. [27], all three participant groups perceived that the pictures supported autistic people to comprehend the meaning of the cards. The pictures were also seen to be important for triggering memories of sensory challenges and eliciting conversations about sensory experiences. Rather than feeling patronised by the use of pictures, tertiary educated and highly articulate autistic people perceived the pictures to be very powerful in stimulating thoughts about sensory experiences. Nevertheless, if any future participants perceived the pictures as being childish, another option is to complete the written response form without accessing the pictures.

The caregiver (proxy‐report) versions were also perceived to be beneficial for people with high support needs. While ideally, we prefer to give the autistic person the opportunity to use the tool independently, we acknowledge that not all autistic people have the capacity to use MYSET independently. Where this is the case, our findings suggest that gaining the perspectives of multiple informants can be helpful. For minimally speaking people, our findings indicated that either the collaborative use of the tool by several people who know the person well, or a hybrid self‐report and proxy‐report approach, made the data gathering process more robust.

Table 3 details our response to feedback about limitations and recommendations for improvement. For the most part, the participants perceived MYSET to be comprehensive. As the length of administration was perceived to be problematic for younger children, the number of cards was reduced by 12%, which substantially reduced the administration time. It was necessary to strike a balance between reducing the length and the addition of suggested new cards. Where possible, existing cards were changed to reflect the autistic participants’ suggestions. The wording on some cards was also simplified to improve accessibility for younger children. For participants who have difficulty making decisions, the MYSET User Manual now suggests that it may be helpful to encourage them to sort the cards based on their first impression or ‘gut reaction’ and to provide assurances that the cards in the “*I’m not sure*” pile will be revisited later. The professional practitioner’s clinical judgement is required to balance giving the person enough time to process information while not encouraging ‘overthinking’, which can make the process excessively lengthy and more stressful. To support participants in rating the impact of the sensory experience, we added a reminder on the Impact Rating Scale to think about whether avoiding a sensory experience is actually a problem in everyday life, irrespective of the intensity of the response. Video‐models to accompany the tool are currently being created, as some participants suggested that this would enhance the explanatory process [56].

**Table 3:** Response to feedback about limitations and recommendations for improvement.

Limitations	Response to feedback
Some considered the tool excessively lengthy especially for younger children.	The number of cards was reduced from 93 to 83 (12% reduction), primarily by amalgamating items (e.g., three cards “*Lots of things in a messy drawer*”, “*Lots of things hanging up in the classroom*” and “*Lots of clutter in an office or workspace*” were amalgamated into one card: “*Mess/clutter [e.g., items on display, things hanging up in classrooms, messy drawer or desk]”*).
Some participants were stressed by their difficulty making decisions about how to sort the cards.	Strategies to reduce stress are detailed in the manual. The manual suggests that it may be helpful to encourage participants who have difficulty making decisions to sort the cards based on their first impression or ‘gut reaction’ with assurances that cards in the “*I’m not sure*” pile can be revisited later.
The wording on some cards was confusing for some participants.	The wording on the cards was reviewed and simplified where possible (e.g., “*Eating unfamiliar foods*” was changed to “*Trying new and different foods*.”).
Impact Rating Scale.	The ‘impact’ takes into account the frequency with which it affects the person’s capacity to do things they need or want to do. We added a reminder to think about whether avoiding a sensory experience is actually a problem in everyday life, irrespective of the intensity of the response (e.g., an individual may detest wearing woolen garments [emotional impact], but as they only ever wear other fabrics [management strategy], the functional impact on daily life is negligible).
Rating of impact on a 10‐point scale required participants to differentiate between the functional impact, emotional impact and management strategy, resulting in uncertainty.	
Recommendations for improvement	Response to feedback
Participants suggested that video‐modelling may further enhance the explanatory process (in addition to the “what to expect stories”)	Video‐models to accompany the tool are being developed
Participants suggested some additional cards	Cards changed to reflect participants suggestions (e.g., an autistic participant suggested a card about “*Light touch …someone putting their hand lightly on your back*”. We replaced two cards: “*Being tapped on the shoulder*” and “*Being bumped”,* with a new card*: “Light or unexpected touch*” that pictured light touch on the back of the neck).

## 5. Limitations and Future Research

Given that MYSET is very new, and that this approach to supporting the sensory challenges and preferences of autistic children and adults has not been trialled before, further research is needed to provide more evidence on the outcomes and usability of the tool. As noted in the method, a limitation was that autistic children and adolescents, and autistic adults with intellectual disability, preferred their parents or professional practitioners to report on their behalf. In future studies, we would ideally like to interview a broader range of autistic children and autistic adults, including people with co‐occurring conditions such as intellectual disability, language disability and specific learning disability, to gain their perspectives on using the tool. Efforts were made to enable non‐speaking people to be involved in the MYSET process to the greatest degree possible (e.g., combining data from two sources by having the non‐speaking person sort the cards independently and having a family member who knows the person well complete the caregiver version). Nevertheless, as it was not possible to engage some people with significant communication and language impairments in using MYSET independently, further research on ways to enhance the participation of people with very high support needs is needed. Although none of the researchers will benefit financially from the sale of MYSET (all proceeds are directed to services for autistic people and their families, and further autism research), a limitation may be that the tool is commercially available which could potentially restrict access for some researchers or participants with limited funding. Future directions may include the development of a digitised version of MYSET, which would enable users to click on text and have it read aloud (i.e., text‐to‐speech function), thus improving accessibility for people with literacy challenges. We would also ideally like to customise the tool for different purposes. For example, versions could be developed to focus more specifically on sensory experiences in schools, workplaces or early childhood settings.

## 6. Conclusion

MYSET is an innovative tool that utilises a picture‐based card‐sort methodology to support conversations with autistic children and adults about their sensory experiences. It generates detailed and highly individualised qualitative data that can be used to collaboratively develop accommodations that are compatible with the person’s lifestyle.

## Funding

No funding was received for this manuscript. Open access publishing facilitated by The University of Queensland, as part of the Wiley ‐ The University of Queensland agreement via the Council of Australasian University Librarians

## Ethics Statement

We obtained ethics approval for each stage of the research from the University of Queensland Human Research Ethics Committee (2009001253; 2022/HE000535).

## Conflicts of Interest

The authors declare no conflicts of interest.

## Supporting Information

Additional supporting information can be found online in the Supporting Information section.Additional supporting information can be found online in the Supporting Information section.

## Supporting information


**Supporting Information 1** Additional supporting information can be found online in the Supporting Information files. The selection of items in MYSET was underpinned by first hand accounts of everyday sensory experiences reported by autistic people, which were sourced through a review of 33 qualitative publications and 5 autobiographies. Supporting Information S1 includes three tables detailing illustrative quotes that correspond to the three MYSET card‐sorting phases as shown in Figure 3. This project complied with both the Standards for Reporting Qualitative Research (SRQR) [45] and the Consolidated Criteria for Reporting Qualitative Research guidelines (COREQ) [46].

**Supporting Information 2** Supporting Information S2 contains the completed SRQR and COREQ checklists, which detail all aspects of the methodology including problem formulation, research questions, background of the researchers, research context, sampling strategy and approach to data analysis.

**Supporting Information 3** Supporting Information S3 contains the checklist used by observers to conduct fidelity observations of the MYSET sessions of the autistic participants. They observed that (1) clear concrete language was used to introduce MYSET, (2) the participants led the card sort, (3) the participants were offered the opportunity to ask questions, (4) the use of the ‘*stop*’, ‘*take a break*’, and ‘*I have a question/I’m confused*’ cards was explained to the participants, (5) the Impact Rating Scale was used by participants to rate the impact of their sensory experiences and (6) the participants’ own words were used on the Strategy and Support Planning and Review sheets.

**Supporting Information 4** Supporting Information S4 includes the semi‐structured interview questions that were used with the autistic participants and family members, and the focus group questions that were used with the professional practitioners or therapy students. The questions covered the participants’ overall impression of the MYSET, ease of understanding of the cards and processes, the comprehensiveness of the cards, helpfulness of the tool in enabling others to understand the person’s sensory experiences, and helpfulness for generating strategies and accommodations that are compatible with their lifestyle.

## Data Availability

Data is not available due to ethical restrictions. (The qualitative data includes information that may result in participants being potentially identifiable.)
