# Supplementary material for: “My Sensory Experiences Tool”: A Neurodiversity‐Affirming Therapeutic Tool to Support the Sensory Challenges and Preferences of Autistic Children and Adults
Source: Occup Ther Int. 2026 Feb 25;2026:4779496. doi: 10.1155/oti/4779496 (PMC12933635; doi:10.1155/oti/4779496)
Supplement: Supplementary file 1 — Supporting Information 1 Additional supporting information can be found online in the Supporting Information files. The selection of items in MYSET was underpinned by first hand accounts of everyday sensory experiences reported by autistic people, which were sourced through a review of 33 qualitative publications and 5 autobiographies. Supporting Information S1 includes three tables detailing illustrative quotes that correspond to the three MYSET card‐sorting phases as shown in Figure 3. This project complied with both the Standards for Reporting Qualitative Research (SRQR) [45] and the Consolidated Criteria for Reporting Qualitative Research guidelines (COREQ) [46]. [file OTI-2026-4779496-s001.docx]

|  | | | |
| --- | --- | --- | --- |
| **Supplementary file S1**  **Table 1:** Illustrative quotes drawn from firsthand accounts in the literature, and correspondence to MYSET Sorting Phase A - Sensitivities and Preferences | | | |
| **Category: Sensory modality** | **Sub-category** | **Illustrative quotes drawn from firsthand accounts in the literature** | **MYSET Cards: LOVE/HATE** |
| **Auditory** | **Heightened reactivity to loud and/or unexpected or unpredictable sounds** | “*Sirens and doorbells make me scared. I cannot concentrate if the noise around me is unpredictable*.” (MacLennan et al., 2022, p. 3070).  *“Both my boys are extremely sensitive to loud, unexpected noise. Fire alarms can be extremely distressing. They become very upset and can cry for long periods of time after the loud noise.”* (Jones et al. 2020, p 6)  *“I’d always shut the door when the hoover* (vacuum clear) *was going. I couldn’t stand the … noise of that.”* (Robertson & Simmons, 2015, p. 579).  *“It* [the sound of balloons] *makes me freak out. I cover my ears. I run like a little girl so afraid*. *Sometimes screaming again covering my ears trying to make it stop*.’’ (Smith & Sharp, 22013, p. 898) | **Loud fireworks** |
|  |  |  | **Loud voices** |
|  |  |  | **Noisy household appliances**  (e.g., blender, vacuum) |
|  |  |  | **Noisy vehicles**  (e.g., trucks, motorbikes) |
|  |  |  | **Noisy bathroom appliances**  (e.g., hand dryers, hair dryers) |
|  |  |  | **Sirens, alarms, school bells** |
|  |  |  | **Sudden loud noises**  (e.g., balloons popping) |
|  | **Distracted by low intensity background noise** | “*Small noises annoy me, like breathing, crunching food or … someone whistling … it makes me ratty*” (Roberton & Simmons 2015, p. 575)  *“In English [class] there was so much noise. I just wanted the class to be quiet, and I can get on with my work.”* (Humphrey & Lewis, 2008).  *“A more common problem is talking—the human voice is not painful but horribly distracting—it ruins my concentration and can be like Chinese water torture”* (Jones et al., 2003, p. 115). | **Background noise when I’m concentrating on something**  (e.g., people talking, typing, radio, TV). |
|  |  |  | **Other people talking** |
|  | **Auditory filtering challenges**  (e.g., sound of someone speaking against competing background noise) | *“Affects concentration on what teacher is saying (i.e. things like peers in room tapping a pen, sliding a ruler across desk, talking or whispering or messing around when he is trying to concentrate on what teacher saying).”* (Jones et al. 2020, p 6).  Some autistic people report that it can be difficult to listen to people talking in a group situation: *“It’s more like a jumble, so many conversations, so I can hear the noise, and I can hear speech but …I can’t isolate one person speaking.”* (Landon et al., 2016. p. 47) | **Listening to the teacher/lecturer in noisy classrooms** |
|  |  |  | **Listening to people talking to them when they are in a group** |
|  | **Auditory preferences/interests**  (e.g., enjoyment of repetitive rhythms or music) | *“I really like music and listen to it as much as I can …I like being able to have full control of what I hear and how loud it is. I’m very specific in the music I listen to and will sometimes just listen to the same band/album/song for weeks.”* (MacLennan et al. 2022, p. 3070)  *““I eventually learned to lose myself in anything I desired… the sound of something over and over again the repetitive hollow sound I’d get from tapping my chin …”* (Williams, 1992, p 3) | **Listening to music​** |
| **Visual** | **Heightened reactivity to bright light** | *“My eyes have struggled in fluorescent light or unnatural light … that sort of stimulus becomes painful after a while.”* (Robertson & Simmons, 2015, p. 575).  *‘‘I particularly dislike bright sunlight. I hate conservatories.”* (Smith & Sharpe, 2023, p. 897)  Participants in a study by Parmar et al. (2021) describe some visual experiences as fatiguing e.g., *“flickering lights, like the sun behind trees, makes me sleepy.”* (p. 6). | **Fluorescent light** |
|  |  |  | **Sunlight** |
|  |  |  | **Lighting in classroom or workplace** |
|  |  |  | **Glare** |
|  |  |  | **Flashing or blinking lights** |
|  | **Visual filtering**  (distracted by visual clutter and/or peripheral movement) | Visually cluttered environments are perceived to be challenging. For example, an autistic student described her dislike of “a *lot of things hanging up*” in her classroom (Zazzi & Faragher, 2018, p. 220).  Visually busy environments can be overwhelming e.g., “*The city centre, being surrounded by buildings and people like it’s all too much visual information*.” (Parmar et al., 2021, p. 6)  Many autistic people report being distracted by movement in their periphery*: “It’s the movement of other people …I’m in my living room, my children running back and forth, I find that very stressful.*” (Parmar et al., 2021, p. 5) | **Mess/clutter messy drawer ​**  (e.g. items on display, things hanging up in the classrooms, messy drawer or desk) |
|  |  |  | **People running around me** |
|  | **Visual preferences/ interests**  (enjoys looking at repetitive patterns, sparkling or shiny objects) | Some autistic people describe their enjoyment of repetitive patterns (e.g., tessellations, floor mats) “*I love how different kinds of patterns are different shapes*.” (Ashburner et al., 2013, p. 175).  *“I absorb myself into the patterns of carpets in unfamiliar settings. It calms the anxiety I feel from being in a new environment.* (Jones et al. 2003, p. 117)  Some enjoy looking at sparkling or shiny objects. For example, Lawson (2000, p. 2) remarked: “*Colour and shiny surfaces are just examples of mediums that connect me to life and to feeling.”* | **Geometric patterns** |
|  |  |  | **Things that sparkle** |
| **Tactile** | **Heightened reactivity to interpersonal touch** | Autistic people frequently over-react to being touched by others, especially when the touch is light or unexpected (e.g., “*It’s dreadful in shops when people bump into you …having to hug strangers who say hello is really nasty*” (Elwin et al., 2013, p, 237).  “*I was once put into ‘‘shellshock’’ for a day-and-a-half because a girl at work tickled me*.” (Jones et al. 2003, p. 116)  A parent expressed concerns about dental visits: *“I thought they were going to sedate him, but the dentist said there are risks involved. My son ended up with a bloody lip and was so agitated. It was traumatic for everyone, including the dentist.”* (Lewis et al., 2015, p E101.) | **Being hugged or kissed** |
|  |  |  | **Light or unexpected touch** |
|  |  |  | **Dentist touching me** |
|  |  |  | **Doctor touching me** |
|  | **Heightened reactivity to touch during self-care tasks** | A parent described the haircutting routine of her autistic child: “W*e stop every time my little one shows distress. My kid plays their tablet and is not expected to sit still. Sometimes the cut isn’t even but we don’t mind.”* (Sterman et al., 2024. P. 241).  *“I struggle with brushing my teeth… I just have to put my toothbrush, even just at the front of my mouth and I’m gagging. Sometimes I actually do vomit as well. It is horrible.”* (Unwin et al., 2025, p. 6) | **Hair cutting or brushing** |
|  |  |  | **Brushing teeth** |
|  |  |  | **Having sunscreen put on** |
|  | **Heightened reactivity to clothing and footwear** | *“Even if I just see it [a wool sweater] hanging in the store, then I already get so itchy on my arms and hands …over my entire upper body.”* (Taels et al., 2023, p. 2274).  “*I only wear cotton next to my skin because of discomfort with how other materials feel*” (Lawson, 2001, p. 181) | **Wearing wool clothes** |
|  |  |  | **Wearing stiff or tight clothes** |
|  |  |  | **Wearing shoes and/or socks** |
|  | **Heightened reactivity to touching certain textures** | Jones et al. (2020) describe *a “child who “really did not like to touch anything that made him ‘dirty’- didn’t like to paint, touch play dough etc. This would make him flap, hit out and sometimes scratch or scream.”*  *“Geetha refused to walk barefoot on grass and sand. She felt so ticklish, and it made her unable to walk.”* (Christopher, 2019, p. 619).  *“I could never take a shower, because I couldn’t stand drops of water on my skin…They had sharp little points that stabbed me. All forms of washing had to happen in the bath.”* (Gerland, 1997, p. 101) | **Touching sticky things** |
|  |  |  | **Touching sand** |
|  |  |  | **Touching grass** |
|  |  |  | **Splashing water**  (e.g., rain, shower, pool). |
|  | **Preference for touching**  **soft textures** | *‘‘Antti is a little cuddly hedgehog I carry around with me… He’s soft. If I’m in a stressful situation and need reassurance I can cuddle him.’’* (Smith & Sharp 2013, p. 900)  *“Love being able to touch soft and squashy things”* (MacLennan et al. 2022, p. 3070). | **Touching soft or furry things** |
| **Olfactory** | **Heightened reactivity to some smells** | *“Smelling a strong smell is like being tortured, time stops and I’m nearly sick.”* (MacLennan et al., 2022, p. 3068).  Autistic school students are reported to be bothered by “*PE changing room*” smells and “*incidental smells such as perfume and cleaning products*” (Jones et al., 2020, p. 6). | **Smell of cleaning products** |
|  |  |  | **Toilet smells** |
|  |  |  | **Body smells** |
|  | **Preferences for/interests**  **in some smells** | Some autistic people enjoy smelling particular odours: *“I wear perfumes a lot because I like fresh scents, and I like clean smells*.” (Singh & Seo, 2022, p. 5)  “*I enjoy using essential oils for scenting the air or massage*.” (Smith & Sharp, 2013, p. 903) |  |
|  |  |  | **Food or cooking smells** |
|  |  |  | **Perfume smells**  (e.g., on other people, in shops etc.) |
| **Movement**  (Vestibular and proprioceptive) | **Heightened reactivity to uncontrollable movement, heights/climbing high** | Some autistic people dislike being moved passively by others or moving when they can’t see where they are going, thus reducing predictability (e.g., dislike of *“moving when I can’t see where I’m going, that’s why when I’m playing Marco Polo with someone, I don’t want to be it.”* (Ashburner et al., 2013, 176).  Fear of heights and climbing up high (elevators, escalators, heights, steps) is common in autistic children. Mayes et al. (2013) found that 18% of 421 autistic children had a fear of heights. | **Moving when can’t see where they are going** |
|  |  |  | **Being upside down** |
|  |  |  | **Being jumped on/tackled** |
|  |  |  | **Climbing up high** |
|  | **Preferences for active movement, difficulty suppressing movement** | Active movement is, by definition, within the person’s control and is therefore predictable. Movement such as “*walking, running or dancing”* is described as a coping strategy to reduce the negative impacts of “*overwhelm*”, “*meltdowns*”, and “*anxiety*” (Petty & Ellis, 2024).  Suppression of movement such as being expected to stand or sit still may be perceived as challenging: “*When I want to move. I’m like, I want to move, I want to move, and if I don’t it’s… [frustrating]*.” (Ashburner et al., 2013, p. 177).  “*He likes to splash in the water. …He just loves water*” (Mische-Lawson et al. 2019, p. 7). | **Standing still** |
|  |  |  | **Sitting still** |
|  |  |  | **Moving in water** |
| **Oral/**  **food sensations** | **Heightened reactivity** to **unfamiliar and/or unpredictable food tastes and textures** | Many autistic people describe over-responsivity to unfamiliar and/or unpredictable food tastes and textures (e.g., “*I didn’t find it dull eating the same thing all the time, though should it start to become so, that was nothing compared to the mortal danger of risking unknown food”* (Gerland, 1997, p. 14).  “*If … [there’s] a strong kind of sensory smell of a food … I couldn’t even think about eating it”, and “[if] textures … were mixed … the sensation makes me want to feel physically sick.”* (Roberts & Simons, 2015, p. 578). | **Eating a variety of food** |
|  |  |  | **Trying new/different food** |
|  |  |  | **Feeling lumpy food in mouth** |
|  |  |  | **Feeling of runny or slippery food in mouth** |
|  |  |  | **Feeling of mixed food in mouth** |
|  | **Preference for a limited range of foods** | Some autistic people fixate on certain foods *(e.g., “I was bulimic for a number of years. This was a form of sensory seeking—I always binged on sweet foods.”* (MacLennan et al., 2022, p. 3068).  Gerland (1997, p. 14) insisted on eating a limited range of foods with smooth textures. *“For long periods, I ate nothing but skinless sausages and chocolate pudding.”* | **Food I eat often** |
| **Multi-sensory** | **Heightened reactivity in multi-sensory environments/situations**  (busy crowded environments and social interactions) | Many autistic people perceive crowded environments with intense multi-sensory input to be especially challenging. For example, supermarkets are characterised by “*Bright unnatural light, shiny floors, visual information from products; noise from checkouts, customers, trolleys, announcements, and background music; smells.*” (MacLennan et al., 2023, p. 419).  *“Some underground train stations have LED advertising screens, and I find them painful to look at because they are too bright …Sometimes, I find that the announcements at train stations are too loud. …On any mode of public transport, there are sometimes people who wear too much perfume or other scented products.”* (Haas et al., 2020, p. 18)  “*Asperger’s made it very difficult for me to cope with life in a large secondary school; there was too much noise and too many people to deal with. It was awful moving to the next class. Everyone was coming out, and it was just swarming with people pushing, running, shoving*.” (Goodall, 2018, p.7).  Participants in a study by Smith & Sharp (2013) described multiple sensory inputs as much more distressing than any single input: “*A lot of people talking in a room, or outside…cars and machinery and birds etc. - just one noise really loud is not as bad.*’’ (p. 898). | **Shopping centre/supermarket/ food hall** |
|  |  |  | **Public transport** |
|  |  |  | **Crowds**  (e.g., shows, concerts, sporting events, conferences) |
|  |  |  | **Crowds at school** |
|  |  |  | **Parties/dinner parties** |
|  |  | Some describe being overwhelmed by the multi-sensory input during social interactions (e.g., eye contact, gestures, tone of voice). ‘‘*I find it very hard to look at a person and listen…well impossible, if I really want to listen*’’ (Smith & Sharp, 2013, p. 900).  Some autistic people “*avoid eye contact or close their eyes when being spoken to in order to reduce the inputs and so avoid being overloaded*.” (Smith & Sharp, 2013, p. 899). | **Eye contact, facial expressions, gestures, and talking** |

**Table 2:** Illustrative quotes drawn from firsthand accounts in the literature, and correspondence to MYSET Sorting Phase B - Seeking/Stimming

| **Category:**  **Sensory modality** | **Sub-category** | **Illustrative quotes drawn from firsthand accounts** | **MYSET Cards: SEEKING and STIMMING** |
| --- | --- | --- | --- |
| Auditory | **Vocal stimming**  Repetitive vocalisations | Gerland (1997, p.30) recalls *“I made sounds in those preschool years – sniffing and grunting – whenever I was doing things. I never noticed them myself, but they disturbed other people.”*  A participant described suppression of his vocal stimming because of other people’s comments: “*When I was very young, I used to hum a lot. At some point, I stopped humming, and I realized that anytime I stopped my stim, it really affected my mental health negatively. Like, when I stopped singing/humming, I got more depressed*.” (Sagar et al., 2023, p. 7). | **Hum or whistle to myself** |
| Tactile | **Tactile stimming**  Repetitive rubbing of soft textures | A participant describes trying to suppress “*stims*” including “*cloth rubbing*” that appeared odd: “*The cloth rubbing, when someone pointed it out to me, I felt that they must be pointing it out to me because it must be odd. So, I stopped doing that …. I had used that stim to help lull me to sleep, so when I stopped doing that stim, I could not sleep. So, that added to my anxiety and depression*” (Sagar et al., 2023, p. 8) | **I feel soft things** |
|  | **Tactile seeking**  Seeking pressure | “*My [boyfriend] knows that if I have a meltdown the best help is to squish me as hard as he can until I feel ok again.*” (MacLennan et al., 2022, p. 3070).  Gerland (1997, p. 5) described seeking deep pressure to help in going to sleep: “*At sleep time, it was best if I could get father to fold the mattress around me, then fasten the sheet firmly around it so that I was lying in the tight roll of the mattress.”* | **Being squished with a pillow** |
| Visual | **Visual stimming**  Watching spinning objects. | An autistic adult remarked that, as a boy, he watched coins spin ‘*seeming never to get bored*.’ (Tammet, 2006, p. 28)  Temple Grandin (1984) commented: “*I had many of the standard autistic behaviours such as fixations on spinning objects*.” | **Look at spinning objects**  (e.g., fans, wheels) |
| Pain | **Self-injurious behaviours** | Goldfarb et al., (2021) found that the main reasons for self-injurious behaviours include: (1) an urge for sensory stimulation and (2) emotional regulation purposes. For example, an autistic adult describes self-pinching for sensory stimulation: “*I don’t do it out of anger, it’s when I feel that my body part is numb, and I need to check if it is functioning or when I got hit . . . I pinch the area to see if I still have a sensation*.” (Goldfarb et al. 2021, p. 1461).  Another autistic adult described self-scratching with a metal wire as a response to emotional pain: “*In difficult times, I had a thing with converting emotional pain to physical pain, really scratching . . . scratching with metal wires until . . . I drew blood . . . It hurt . . . It was severe violence toward myself, as a way of substituting mental pain with physical . . . It indicates very, very, very, very strong emotional distress*” (Goldfarb et al. 2021, p. 1461). | **Hurt myself**  (e.g., scratch, pull hair, bang head, bite) |
| Olfactory | **Seeking**  **of particular smells** | Some autistic people with smell sensitivity insist on smelling food before eating it: “*If it does not smell good, there is no way I’m going to eat it.*” (Singh & Seo, 2021, p, 4).  Pillar and Pfeiffer (2016, p. 106) describe a child who constantly smelled markers to the point that he would not use them to colour or write. “*He would request them [markers] a lot and he would just sit there smelling them.”* | **Smell food**  (e.g., will smell food before eating) |
|  |  |  | **Smell things**  (e.g., plants, soap, perfume) |
| Oral sensations | **Oral seeking/mouthing of non-food substances** | Mouthing of non-food substances is frequently reported. Parents often describe their autistic children as “*loving to chew things*” (Dickie et al., 2009).  “*She (autistic child) moved from being selective in her choice of foods to biting every hard object she stumbled upon.”* (Masiran, 2018, p. 1).  Gerland (1997, p. 15) said “*I liked biting people, and on the odd occasion I was allowed to bite my sister. But mostly I had to be content with things made of soft plastic – my old teething ring, toys, furniture ... whenever I had to calm that unpleasant feeling in my teeth.”* | **Chew or suck objects**  (e.g., pens, shirt, hair) |
| Movement (vestibular & proprioceptive) | **Stimming behaviours involving movement**  (repetitive hand movement, rocking, spinning, pacing, jumping, swinging etc.) | Some autistic people describe stimming as helping with emotional regulation: “*Usually to relieve a build-up of feelings before I get overwhelmed. Flapping and singing are when I'm excited, finger flicking when anxious*” (Charlton et al., 2021, p. 5).  A participant in a study by Sagar et al. (2023, p. 8) commented “*I rock myself to sleep every time. When I am nervous or anxious … I stim. When I am happy, I stim … Extreme anger or helplessness also trigger me to stim.”*  The autistic adolescents in the study by Ashburner et al. (2013) engaged in a range of repetitive movement activities such as jumping on the trampoline and spinning: “*I strap myself into a spinning chair and spin around… [for] two good hours*.”  A participant in the study by Kapp et al. (2019, p. 1788) said: *“I* *remember as a child spinning all the time and loving spinning and loving swinging …I also realised that there was a point where it wasn’t acceptable to be spinning anymore … so it actually still feels glorious if there’s nobody around and … I can spin*.”  A parent told how *“before we knew he was autistic, he bounced and bounced and bounced* …” (Dickie et al. 2009, p. 177).  Repetitive movements such as rocking, pacing, walking, waving arms, stroking the body or jiggling have been described by autistic authors as providing *“comfortable background signal”* that helped them to ignore distractions in overwhelming or noisy environments (Petty & Ellis, 2024, p. 3018), | **Move hands over and over**  (e.g., shaking, flapping, clapping) |
|  |  |  | **Rock back and forth** |
|  |  |  | **Spin**  (e.g., on an office chair) |
|  |  |  | **Walk or run back and forth** |
|  |  |  | **Jump**  (e.g., on a trampoline) |
|  |  |  | **Swing**  (e.g., on a swing or hammock) |
|  |  |  | **Jiggle my legs** |

**Table 3:** Illustrative quotes drawn from firsthand accounts in the literature and correspondence to MYSET Sorting Phase C - Awareness of Sensory Input

| **Category: Sensory modality** | **Subcategory** | **Illustrative quotes drawn from firsthand accounts in the literature** | **MYSET Cards:**  **NOTICING SENSATIONS** |
| --- | --- | --- | --- |
| **Interoception** | **Decreased or increased reactivity to internal body sensations** | *“I cannot consistently tell when I am hungry. My brain doesn’t alert me until a very high threshold of hunger has been reached. I eat according to the clock, not the hunger signal. The only time I can tell when I am hungry is when I have not eaten for a whole day or longer*.” (Trevisan et al. 2021, p, 3485). | **Hunger and thirst** |
|  |  | *“I often say, because everyone is staring at me and I am not wearing very much, that there is something wrong with my heating system … coz when others are wearing four layers of clothes, I might have one … I don’t react to heat or cold either.” (Elwin et al., 2013, p. 236)* | **Feeling hot**  (e.g., hot summer day) |
|  |  |  | **Feeling cold**  (e.g., cold winter day) |
|  |  | “…*others always have to tell me that I look like I am in very bad shape or having serious symptoms, because I don’t even notice it*.” (Trevisan et al. 2021, p, 3486).  An autistic participant described that sometimes when she cut herself, she would not feel pain. “*I didn’t feel the skin being pierced because I don’t have normal feeling in my skin*.” (Robledo et al. 2012, p. 6).  “*When I’m really sad, it physically hurts. The best way I can describe it as it’s like my whole-body stings very, very badly or is on fire. …Maybe I’m noticing subtle changes in my body that most people aren’t aware of and experiencing them as painful.*” (Trevisan et al. 2021, p, 3486). | **Pain** |
|  |  | Some autistic people describe reduced awareness of their own emotional state, which they attribute to reduced sensitivity to internal sensations such as heart or breathing rate. *“I think my inability to identify emotional states in myself is fundamentally sensory disorder. I understand that people have specific sensations (feelings) in their bodies when they are experiencing a particular emotion. However, I have many sensations in my body that are not associated with emotional context”* (Trevisan et al. 2021, p, 3487). | **Heart rate and breathing**  (e.g., when stressed or doing physical exercise) |
|  |  | “…*I’m insensitive to my body’s signal that I need to go…I’m very often extremely desperate to go once I finally realise and so can have a bit of* [an] *accident when I move to get to the toilet, or if there isn’t one handy, because I’m so close to bursting already.”* (Trevisan et al., 2021, p. 3486).  *“I had this problem for several years* (incontinence)*. I was, as you say, too absorbed to pay attention to what my body was telling me. And I’m pretty sure that most of the time having to go just didn’t register.”* (Trevisan et al., 2021, p. 3486). | **Needing to use the toilet** |
| **Managing attentiveness to sensory stimuli** | **Failure to respond when over-focused** | Participants in the study by Ashburner et al. (2013, p. 175) described not noticing others talking to them when overfocusing on something e.g. “*Mum yells ‘Luke’. I’m like what*?”  “*When I watch Formula One on TV, I get completely absorbed in it and I wouldn’t even notice if … a bomb went off next to me*.” (Taels et al., 2023, p. 2275) | **People talking to me when I am concentrating on something** |
|  | **Overfocusing on extraneous details** | Some autistic people describe a tendency to notice small or unusual aspects of stimuli that other people don’t notice (e.g., “*I notice sounds made by the fridge …that the staff don’t notice at all, but I ask them like what is making that noise, and they can’t hear it*” (Elwin et al., 2013, p. 236).  A participant in a study by Landon et al. (2016, p. 46) was irritated by background humming sounds: *“Definitely electronic things, like the computer and the buzzing light umm . . . umm buzzing fluorescent light, umm ticking, clocks, …things that are kind of quiet and insidious*.”  *“I had a fluorescent light in my room, the buzzing was so annoying that it got to the point where I couldn’t turn it on. So, I sat there in the dark in my room for half the year*…” (Landon et al. p, 48). | **Humming sounds from electrical appliances** (e.g., lights, air conditioner, fridge) |

**References**

Ashburner, J., Bennett, L., Rodger, S., & Ziviani, J. (2013). Understanding the sensory experiences of young people with autism spectrum disorder: A preliminary investigation. *Australian Occupational Therapy Journal*, *60*(3), 171-180. [https://doi.org/[10.1111/1440-1630.12025](https://doi.org/10.1111/1440-1630.12025)](https://doi.org/)

Charlton, R. A., Entecott, T., Belova, E., & Nwaordu, G. (2021). “It feels like holding back something you need to say”: Autistic and non-autistic adults accounts of sensory experiences and stimming. *Research in Autism Spectrum Disorders*, *89*, 101864. <https://doi.org/10.1016/j.rasd.2021.101864>

Christopher, S. (2019). Touch hypersensitivity in children with autism – An analysis. *International Journal of Research and Analytical Reviews*, *6*(2), 616-622.

Dickie, V. A., Baranek, G. T., Schultz, B., Watson, C. R. & McCormish, C. S. (2009). Parent reports of sensory processing experiences of preschool children with and without autism: A qualitative study. The American Journal of Occupational Therapy, 63(2), 172–181. <https://doi.org/10.5014/ajot.63.2.172>

Elwin, M., Ek, L., Kjellin, L., & Schröder, A. (2013). Too much or too little: Hyper-and hypo-reactivity in high-functioning autism spectrum conditions. *Journal of intellectual & developmental disability*, *38*(3), 232-241. https://doi.org/[10.3109/13668250.2013.815694](https://doi.org/10.3109/13668250.2013.815694?urlappend=%3Futm_source%3Dresearchgate.net%26utm_medium%3Darticle)

Gerland, G. (1997). *A real person – Life on the Outside*. London Souvenir Press.

Goodall, C. (2018). Mainstream is not for all: The educational experiences of autistic young people. *Disability & Society*, *33*(10), 1661-1665. <https://doi.org/10.1080/09687599.2018.1529258>

Goldfarb, Y., Zafrani, O., Hedley, D., Yaari, M., & Gal, E. (2021). Autistic adults’ subjective experiences of hoarding and self-injurious behaviors. *Autism*, *25*(5), 1457-1468. [https://doi.org/10.1177/1362361321992640](https://psycnet.apa.org/doi/10.1177/1362361321992640)

Grandin, T. (1984). My experiences as an autistic child and review of selected literature. *Journal of orthomolecular psychiatry*, *13*(3), 144-174.

Haas, K., Wilson, N.J., Cordier, R., Vaz, S. & Chung-yeung Lee, H. (2020). *The experiences of young autistic adults in using metropolitan public transport.* Brisbane, Australia: Cooperative Research Centre for Living with Autism

Humphrey, N., & Lewis, S. (2008). ‘Make me normal' The views and experiences of pupils on the autistic spectrum in mainstream secondary schools. *Autism*, *12*(1), 23-46. <https://doi.org/10.1177/1362361307085267>

Jones, E. K., Hanley, M., & Riby, D. M. (2020). Distraction, distress and diversity: Exploring the impact of sensory processing differences on learning and school life for pupils with autism spectrum disorders. *Research in autism spectrum disorders*, *72*, 101515. <https://doi.org/10.1016/j.rasd.2020.101515>

Jones, R. S., Quigney, C., & Huws, J. C. (2003). First-hand accounts of sensory perceptual experiences in autism: A qualitative analysis. *Journal of Intellectual & Developmental Disability*, *28*(2), 112-121. <https://doi.org/10.1016/j.rasd.2020.101515>

Kapp, S. K., Steward, R., Crane, L., Elliott, D., Elphick, C., Pellicano, E., & Russell, G. (2019). ‘People should be allowed to do what they like’: Autistic adults’ views and experiences of stimming. *Autism*, *23*(7), 1782-1792. <https://doi.org/10.1177/1362361319829628>

Landon, J., Shepherd, D., & Lodhia, V. (2016). A qualitative study of noise sensitivity in adults with autism spectrum disorder. *Research in Autism Spectrum Disorders*, *32*, 43-52. https://doi.org/[10.1016/j.rasd.2016.08.005](https://doi.org/10.1016/j.rasd.2016.08.005)

Lawson, W. (2000). *Life behind glass: A personal account of autism spectrum disorder*. London: Jessica Kingsley Publishers.

Lawson, W., (2001). *Understanding and working with the spectrum of autism: An Insider’s View*. London: Jessica Kingsley Publishers.

Lewis, C., Vigo, L., Novak, L., & Klein, E. J. (2015). Listening to parents: A qualitative look at the dental and oral care experiences of children with autism spectrum disorder. *Pediatric dentistry*, *37*(7), 98E-104E.

MacLennan, K., O’Brien, S., & Tavassoli, T. (2022). In our own words: The complex sensory experiences of autistic adults. *Journal of Autism and Developmental Disorders*, *52*(7), 3061-3075. <https://doi.org/10.1007/s10803-021-05186-3>

MacLennan, K., Woolley, C., @ 21andsensory, E., Heasman, B., Starns, J., George, B., & Manning, C. (2023). “It is a big spider web of things”: Sensory experiences of autistic adults in public spaces. *Autism in Adulthood*, *5*(4), 411-422. https://doi.org/[10.1089/aut.2022.0024](https://doi.org/10.1089/aut.2022.0024)

Masiran, R. (2018). Stimming behaviour in a 4-year-old girl with autism spectrum disorder. *Case Reports*, *2018*, bcr-2017.

Mayes, S. D., Calhoun, S. L., Aggarwal, R., Baker, C., Mathapati, S., Molitoris, S., & Mayes, R. D. (2013). Unusual fears in children with autism. *Research in Autism Spectrum Disorders*, *7*(1), 151-158. https://doi.org/[10.1016/j.rasd.2012.08.002](https://doi.org/10.1016/j.rasd.2012.08.002)

Mische Lawson, L., D’Adamo, J., Campbell, K., Hermreck, B., Holz, S., Moxley, J., ... & Travis, A. (2019). A qualitative investigation of swimming experiences of children with autism spectrum disorders and their families. *Clinical Medicine Insights: Pediatrics*, *13*, 1179556519872214. https://doi.org/[10.1177/1179556519872214](https://doi.org/10.1177/1179556519872214)

Parmar, K. R., Porter, C. S., Dickinson, C. M., Pelham, J., Baimbridge, P., & Gowen, E. (2021). Visual sensory experiences from the viewpoint of autistic adults. *Frontiers in Psychology*, *12*, 633037. https://doi.org/[10.3389/fpsyg.2021.633037](https://doi.org/10.3389/fpsyg.2021.633037)

Petty, S., & Ellis, A. (2024). The meaning of autistic movements. *Autism*, *28*(12), 3015-3020. [https://doi.org/10.1177/136236132412621](https://doi.org/10.1177/13623613241262151)

Piller, A., & Pfeiffer, B. (2016). The sensory environment and participation of preschool children with autism spectrum disorder. *OTJR: occupation, participation and health*, *36*(3), 103-111. <https://doi.org/10.1177/1539449216665116>

Robertson, A. E., & Simmons, D. R. (2015). The sensory experiences of adults with autism spectrum disorder: A qualitative analysis. *Perception*, *44*(5), 569-586. <https://doi.org/10.1068/p7833>

Robledo, J., Donnellan, A. M., & Strandt-Conroy, K. (2012). An exploration of sensory and movement differences from the perspective of individuals with autism. *Frontiers in Integrative Neuroscience*, *6*, 107. <https://doi.org/10.3389/fnint.2012.00107>

Sagar, E., Khera, S. N., & Garg, N. (2024). “I Wish They'd Just Let Us Be.” Experiences of Indian autistic individuals around stimming behaviors at the workplace. *Autism in Adulthood, 6*(4), 474-484. [https://doi.org/10.1089/aut.2022.0](https://doi.org/10.1089/aut.2022.0096)

Singh, A., & Seo, H. S. (2022). Atypical sensory functions and eating behaviors among adults on the autism spectrum: One‐on‐one interviews. *Journal of Sensory Studies*, *37*(2), e12724 https://doi.org/[10.1111/joss.12724](https://doi.org/10.1111/joss.12724?urlappend=%3Futm_source%3Dresearchgate.net%26utm_medium%3Darticle)

Smith, R. S., & Sharp, J. (2013). Fascination and isolation: A grounded theory exploration of unusual sensory experiences in adults with Asperger syndrome. *Journal Of Autism and Developmental Disorders*, *43*(4), 891-910. https://doi.org/[10.1007/s10803-012-1633-6](https://doi.org/10.1007/s10803-012-1633-6)

Sterman, J., Gustafson, E., Eisenmenger, L., Hamm, L., & Edwards, J. (2023). Autistic adult perspectives on occupational therapy for autistic children and youth. *OTJR: Occupational Therapy Journal of Research*, *43*(2), 237-244. https://doi.org/[10.1177/15394492221103850](https://doi.org/10.1177/15394492221103850?urlappend=%3Futm_source%3Dresearchgate.net%26utm_medium%3Darticle)

Taels, L., Feyaerts, J., Lizon, M., De Smet, M., & Vanheule, S. (2023). ‘I felt like my senses were under attack’: An interpretative phenomenological analysis of experiences of hypersensitivity in autistic individuals. *Autism*, *27*(8), 2269-2280. [https://doi.org/10.1177/136236132311581](https://doi.org/10.1177/13623613231158182)

Tammet, D. (2006). *Born on a blue day*. London: Hodder.

Trevisan, D. A., Parker, T., & McPartland, J. C. (2021). First-hand accounts of interoceptive difficulties in autistic adults. *Journal of Autism and Developmental Disorders*, 51(10), 3483-3491. https://doi.org/[10.1007/s10803-020-04811-x](https://link.springer.com/article/10.1007/s10803-020-04811-x)

Unwin, K. L., Philpott-Robinson, K., Barbaro, J., Dwyer, P., Sadka, N., Date, P., & Lane, A. E. (2025). 'We're quite good at thinking outside the box: Early autistic sensory experiences expressed by autistic adults and caregivers of autistic children. *Autism: The international journal of research and practice*, 13623613251335069. Advance online publication. https://doi.org/10.1177/13623613251335069

Williams, D. (1992). *Nobody Nowhere.* London, Doubleday

Zazzi, H., & Faragher, R. (2018). ‘Visual clutter’ in the classroom: Voices of students with autism spectrum disorder. *International Journal of Developmental Disabilities*, *64*(3), 212-224. https://doi.org/10.1080/20473869.2018.1468619
