# Supplementary material for: “My Sensory Experiences Tool”: A Neurodiversity‐Affirming Therapeutic Tool to Support the Sensory Challenges and Preferences of Autistic Children and Adults
Source: Occup Ther Int. 2026 Feb 25;2026:4779496. doi: 10.1155/oti/4779496 (PMC12933635; doi:10.1155/oti/4779496)
Supplement: Supplementary file 2 — Supporting Information 2 Supporting Information S2 contains the completed SRQR and COREQ checklists, which detail all aspects of the methodology including problem formulation, research questions, background of the researchers, research context, sampling strategy and approach to data analysis. [file OTI-2026-4779496-s002.docx]

**Supplementary File S2:**

**Standards for Reporting Qualitative Research (SRQR):**

**Completed SRQR checklist (O’Brien et al., 2014)**

**Title and abstract**

***S1 Title.*** The title does not include information on the research methodology, but the fact that it is a qualitative exploration is indicated in the second paragraph in the introduction on page 4:

This paper first describes an innovative therapeutic tool called the My Sensory Experiences Tool (MYSET®), which aims to provide an accessible way for autistic people to explain their experiences of their sensory world. The considerations that guided the development of the tool are also outlined. Secondly, the paper explores the perceptions of autistic people, family members and professional practitioners of the usefulness of MYSET® and ways it could be improved.

It was not possible to include this in the title without leaving our other essential information about what MYSET^®^ is.

***S2 Abstract.*** The abstract includes the following key elements: Background, Purpose, Methods, Findings, and Conclusions. **(page 3).**

**Introduction (pages 4-7)**

***S3 Problem formulation.*** The introduction positions the phenomenon being studied in the context of previous approaches to sensory processing information gathering. This includes an analysis of the gaps in current sensory processing information gathering approaches, and an overview of the considerations that guided the development of MYSET^®^. The latter includes the theoretical alignment with a neurodiversity-affirming paradigm, accessibility to a diversity of autistic people, and the approach used to select the items in MYSET^®^.

***S4*** ***Purpose or research question.*** The research purpose and research questions are clearly stated on **page 20** as follows:

***Research aim:***

*The study aimed to explore the perspectives of autistic people, family members and professional practitioners on the usefulness of MYSET*^®^ *and to explore ways that it could be improved.*

***Research questions****:*

*• How do autistic people, family members and professional practitioners perceive the usefulness of the information gained using MYSET®?*

*• How do autistic people, family members and professional practitioners perceive the utility of MYSET® processes, including the what-to-expect stories, the card-sorting, the Impact Rating Scale and the Strategy and Support Planning Process?*

*• Do autistic people, family members and professional practitioners perceive MYSET® to be helpful in enabling others in the person’s life to understand their sensory experiences?*

*• Do autistic people, family members and professional practitioners perceive MYSET® to be helpful in enabling the generation of strategies and accommodations that are compatible with the autistic person’s lifestyle?* **(Page 20)**

**Method**

***S5 Qualitative approach and research paradigm:*** A qualitative descriptive approach was used within a constructivist paradigm emphasising the participants’ subjective experiences (Creswell, 2014). Inductive content analysis, which is a systematic method used to identify, organise and interpret patterns in qualitative data, was used understand to analyse the data. Inductive content analysis was considered appropriate because this study aimed to explore specific and practical perspectives on using the tool in clinical practice without imposing predefined categories (Vears & Gillam, 2022**). (Page 20)**

***S6 Researcher characteristics and reflexivity****.* JA, VT and CD all have experience in conducting qualitative research (4-18 years’ experience) in addition to being allied health professionals with experience in working with autistic people (4-30 years’ experience). **(Page 24)**

*Research qualifications and experience:*

- JA has a Bachelor of Occupational Therapy and a PhD focusing on sensory processing in autism, 30 years’ experience as an occupational therapist including multiple senior roles in addition to 18 years’ experience in autism research including multiple projects focusing on sensory processing.
- VT has a Bachelor of Occupational Therapy (Hons), 14 years’ experience as an occupational therapist including multiple senior roles and 4 years research officer experience including playing a major role in the development of a goal setting tool for autistic people.
- CD has a Bachelor of Science/Bachelor of Arts, a Master of Audiological studies, and a PhD. She has 4 years’ experience as an audiologist and 5 years’ research officer experience including a project focusing on auditory processing of autistic children.

*Relationship with participants*. Some of the participants including the majority of the professional practitioners, some of the autistic participants and some of the family member participants were known to the interviewers. As experienced allied health professionals, the interviewers were able to readily establish rapport with the other participants during the initial contact to arrange a time for the interview.

*Researcher positionality* **(page 27).** JA, VT, JH and JC all have an occupational therapy background which shaped the daily life participation focus of MYSET^®^ (Vessby & Kjellberg, 2010). JA and VT occupied an insider position, as they were responsible for the development of MYSET™, giving insight into the rationale behind aspects of the MYSET^®^ design. The risk of bias was mitigated by engaging researchers from a local university, JH and JC, who provided an outsider perspective by reviewing and questioning the interpretation of the data through processes such as double coding.

***S7 Context****:* The contextual factors that influenced the research was that it was positioned within a large service provider organization for autistic children and adults (see Development of MYSET^®^ on **page 8**). The professional practitioners had used MYSET^®^ with participants with a range of ages (children and adults) and communicative abilities. As shown on **page 21** and in *Table 1: Demographics of professional practitioners* on **Page 25**, they had also used the tool in a range of service contexts including service areas, including individual therapy, positive behaviour support, autism-specific schools, mainstream school consultancy, mental health services, supported accommodation, and assessment and diagnostic services. This enabled us to explore the applicability of the tool to different contexts and to clients with diverse characteristics.

***S8 Sampling strategy.*** The professional practitioners were purposively sampled to include practitioners working in a range of service areas, including individual therapy, positive behaviour support, autism-specific schools, mainstream school consultancy, mental health services, supported accommodation, and assessment and diagnostic services. We also aimed to recruit professional practitioners seeing clients with a range of cognitive/communication abilities. The 18 professional practitioners had used MYSET^®^ with 41 autistic clients aged 5-49 years, including five with a suspected cognitive/language impairment, and seven with a diagnosed intellectual disability. The autistic participants were purposively sampled to include a range of ages (10-54 years). The family members were purposively sampled to include parents of children with a range of communication abilities (ranging from age-appropriate communication skills to minimally speaking). **(See section on Participants on pages 21-22.)**

***S9 Ethical issues pertaining to human subjects.*** We obtained ethics approval from the ***[removed for peer review]*** Human Research Ethics Committee ***[removed for peer review]*.** All autistic adults, professional practitioners and parents of autistic children provided informed written consent, while autistic children provided written assent. Autistic participants received an ‘*Easy English’* version of the participant information sheet with picture cues. **(See Ethical Considerations on Page 20.)**

***S10 Data collection method.*** Qualitative data was gathered through semi-structured interviews with the autistic participants and family members, and focus groups with the professional practitioners, conducted via Microsoft Teams® videoconferencing. Data was collected between February and December 2023. The data was triangulated by gaining the perspectives of multiple stakeholder groups (autistic people, their family members and professional practitioners). **(See section on Data Collection on pages 23-24.)**

***S11 Data collection instruments and technologies.*** The semi-structured interview guides for the autistic participants and family members and focus group questions for the professional practitioners are shown in Supplementary file C. The interview and focus groups were conducted via Microsoft Teams® videoconferencing. **(See section on Data Collection on pages 23-24.)** With the aim of supporting transparency and the interpretation of the participant’s quotes (O’Brien et al., 2014; Tong et al., 2007), the characteristics of autistic participants likely to impact on their functional capacity were assessed. The assessments included the *Social Responsiveness Scale* *(2^nd^ Edition)* (SRS-2) (Constantino, & Gruber, 2012), the *Adaptive Behaviour Assessment Scale* *(3^rd^ edition)* (ABAS-3) (Harrison & Oakland, 2015), and the *Kaufman Brief Intelligence Test* *(2^nd^ edition)* (K-BIT-2) (Kaufman & Kaufman, 2004). These assessments were used under license from their respective publishers (Pearson for the K-BIT-2 and ABAS-3, Western Psychological Services for the SRS-2) and administered by qualified personnel in accordance with the publisher guidelines. Their use was approved by the ***[removed for peer review]*** Human Research Ethics Committee ***[removed for peer review]*** as part of the approved research protocol. **(See section on Participants on page 22.)**

***S12 Units of study.*** Eighteen professional practitioners, five autistic individuals and four family members were recruited through the networks of ***[removed for peer review]*** a service provider for autistic people, according to the following criteria: (a) professional practitioners who had facilitated the use of MYSET^®^, (b) autistic participants who had used MYSET^®^, and (c) family members who had supported their child in using MYSET^®^. **(See section on Participants on page 21.)**

***S13 Data processing.*** The written transcripts generated by the video-conferencing platform were reviewed for accuracy and anonymised before analysis. All transcripts were read thoroughly to enhance familiarity with the data. JA manually coded the data into subcategories, and categories, and developed a preliminary coding dictionary with clear descriptions of each code. A coding tree (codes, code descriptors, sub-categories and categories) is provided at the end of this document in Supplementary File S2 Table 1. JH, who provided an outsider perspective, independently re-coded 100% of the data. **(See section on Data Analyses on pages 27-28.)**

***S14 Data Analysis.*** We analysed the data using inductive content analysis (Kyngas, 2020). Inductive content analysis was considered appropriate because this study aimed to explore specific and practical perspectives on using the tool in clinical practice without imposing predefined categories (Vears & Gillam, 2022). Using a consensus coding method. JA and JH met to review, discuss and collaboratively refine the codes until they reached agreement on the final codes (Cascio et al. 2019). **(See section on Data Analyses on pages 27-28.)**

***S15 Techniques to enhance trustworthiness.*** Strategies to improve trustworthiness included: (a) triangulation by gaining the perspectives of multiple stakeholder groups, (b) having two coders independently review the findings, and (c) a member checking procedure which involved one autistic person (AP01), one family member (FM04) and one professional practitioner (PP08) reviewing the synthesised and analysed data. The latter participants were selected as member checkers, as they had been involved in the most recent interviews or focus groups and were thus the most likely to recall the details of the discussion. The member checkers agreed that the way that their comments had been coded accurately represented their experiences. **(See section on Data Analyses on pages 27-28.)**

**Results/Findings**

***S16 Synthesis and interpretation.*** The findings comprised six categories and three sub-categories within category 1. The information in the black squares in Figure 4 shows the key features and outcomes of MYSET^®^, while the information in the grey squares shows participant perceptions of limitations and suggested improvements. Category 1 describes the key features of MYSET^®^ which includes the following three sub-categories: (a) the nature of the information gathered, (b) the tool’s accessibility to a diversity of autistic people, and (c) the MYSET^®^ processes. These key features enhance the autistic person’s capacity to self-reflect on their own sensory experiences (Category 2) and enable them to share their experiences with others (Category 3). Both the person’s self-reflection and other’s understanding of their sensory experiences enable the development of customized reports on effective strategies and support planning (Category 4). The information in the grey squares in Figure 4 shows two categories including participant feedback on limitations to be addressed (Category 5) and suggested improvements (Category 6). **(See Figure 4 on page 29 and section on Findings on pages 30-37.)**

***S17 Links to empirical data.*** The findings are illustrated using direct participant quotes. Participants were assigned identifier numbers corresponding to their participant group (autistic participants = AP, family member = FM, professional practitioner = PP). **(See section on Findings on page 30.)**

**Discussion**

***S18 Integration with prior work, implications, transferability and contributions to the field***. The findings are discussed in the context of previous literature on sensory processing information gathering approaches (Blackwell et al., 2023; Dubois et al., 2017; Lucas et al., 2024; McLennan et al., 2022). The relationship of MYSET^®^ to theoretical positions on neurodiversity affirming principles (Pellicano & den Houting, 2021) is also discussed. With respect to generalisability, we have addressed the accessibility of MYSET^®^ to a diversity of autistic people. In terms of contribution to the field, the approach taken by MYSET^®^ is highly innovative in that this approach has not been previously used in clinical practice. **(See section on Discussion on page 37-41.)**

***S19 Limitations.*** Given that MYSET^®^ is very new, and that this approach to supporting the sensory challenges and preferences of autistic children and adults has not been trialed before, future research needs to provide more evidence of positive outcomes and useability of the tool. As noted in the method, a limitation was that autistic children and adolescents, and autistic adults with intellectual disability, preferred their parents or professional practitioners to report on their behalf. In future studies, we would ideally like to interview a broader range of autistic children and autistic adults, including people with co-occurring conditions such as intellectual disability, language disability, and specific learning disability to gain their perspectives on using the tool. Efforts were made to enable non-speaking people to be involved in the MYSET^®^ process to the greatest degree possible (e.g., combining data from two sources by having the non-speaking person sort the cards independently and having a family member who knows the person well complete the caregiver version). Nevertheless, as it was not possible to engage some people with significant communication and language impairments in using MYSET^®^ independently, further research on ways to enhance the participation of people with very high support needs is needed. Although none of the researchers will benefit financially from the sale of MYSET® (all proceeds are directed to services for autistic people and their families), a limitation may be that the tool is commercially available which could potentially restrict access for some researchers or participants with limited funding. **(See section on Limitations and Future Research on page 41-42.)**

**Consolidated Criteria for reporting qualitative research (COREQ) and Standards for Reporting Qualitative Research (SRQR):**

**Completed COREQ Checklist** (Tong et al., 2007)

**Domain 1: Research team and reflexivity**

**Personal characteristics**

1. *Interviewer:* The interviews were conducted by JA, VT, and CD
2. *Credentials:*

- JA has a Bachelor of Occupational Therapy and a PhD focusing on sensory processing in autism, 30 years’ experience as an occupational therapist including multiple senior roles in addition to 18 years’ experience in autism research including multiple projects focusing on sensory processing.
- VT has a Bachelor of Occupational Therapy (Hons), 14 years’ experience as an occupational therapist including multiple senior roles and 4 years research officer experience including playing a major role in the development of a goal setting tool for autistic people.
- CD has a Bachelor of Science/Bachelor of Arts, a Master of Audiological studies, and a PhD. She has 4 years’ experience as an audiologist and 5 years’ research officer experience including a project focusing on auditory processing of autistic children.

1. *Occupation:* During this project:
   - JA was employed as a Manager Research and Development.
   - VT was employed as a Research Development and Commercialisation Officer.
   - CD was employed as a Senior Research Officer.
2. *Gender:* JA, VT and CD identify as female.

*Experience and training:* JA, VT and CD all have experience in conducting qualitative research (4-18 years’ experience) in addition to being allied health professionals with experience in working with autistic people (4-30 years’ experience). **(See section on Data collection pertaining to conducting interviews on page 24.)**

**Relationship with participants**

1. *Relationship established:* Some of the participants including the majority of the professional practitioners, some of the autistic participants and some of the family member participants were known to the interviewers. As experienced allied health professionals, the interviewers were able to readily establish rapport with the other participants during the initial contact to arrange a time for the interview. The study aims and processes were explained clearly to all participants. To inform the autistic children of what to expect, a Social Story™ (Gray, 2010) and a visual schedule were used. A visual ‘stop’ and a ‘take a break’ card were available to all participants to use if they wished to halt the interview or withdraw from the study (Harrington et al., 2013). Additionally, the researchers altered their vocabulary to match the participant’s communication style, so as to make the questions more meaningful to them.
2. *Participant knowledge of the interviewer:* All participants were aware that JA, VT and CD were researchers on this project, and that they had no direct involvement in delivering services that could impact on the supports that the participants receive.
3. *Interviewer characteristics***:** JA, VT and CD did not have any conflicts of interest that could bias the way that the interviews were conducted. None of the researchers has any financial interest in the project. As all proceeds from the sale of MYSET^®^ were directed to services for autistic people. JA and VT have an occupational therapy background which influenced the daily life participation focus of the tool (Vessby & Kjellberg, 2010). **(See section on Data collection pertaining to conducting interviews on page 24.)**

**Domain 2: study design**

**Theoretical framework**

1. *Methodological orientation and theory:* A qualitative descriptive approach was used within a constructivist paradigm emphasising the participants’ subjective experiences (Creswell, 2014). Inductive content analysis, which is a systematic method used to identify, organise and interpret patterns in qualitative data, was used understand to analyse the data. Inductive content analysis was considered appropriate because this study aimed to explore specific and practical perspectives on using the tool in clinical practice without imposing predefined categories (Vears & Gillam, 2022). **(See section on Data analysis section on page 27.)**

**Participant selection:**

1. *Sampling:* The professional practitioners were purposively sampled to include practitioners working in a range of service areas, including individual therapy, positive behaviour support, autism-specific schools, mainstream school consultancy, mental health services, supported accommodation, and assessment and diagnostic services. We also aimed to recruit professional practitioners seeing clients with a range of cognitive/communication abilities. The 18 professional practitioners had used MYSET^®^ with 41 autistic clients aged 5-49 years, including five with a suspected cognitive/language impairment, and seven with a diagnosed intellectual disability. The autistic participants were purposively sampled to include a range of ages (10-54 years). The family members were purposively sampled to include parents of children with a range of communication abilities (ranging from age-appropriate communication skills to minimally speaking). **(See section on Data analysis section on page 27.)**
2. *Method of approach:* The participants recruited through the networks of ***[removed for peer review]*** a large service provider for autistic children and adults. They were all contacted via email to ask about potential participation.
3. *Sample size:* Eighteen professional practitioners, five autistic individuals, and four family members were recruited.
4. *Non-participation:* One autistic woman and her autistic child withdrew from the study due to time constraints. **(See section on Participants on page 21-22.)**

**Setting**

1. *Setting of data collection:* Data was gathered through video-recorded sessions *(Microsoft Teams™* meetings) with the interviewees being at home. The MYSET^®^ was administered face-to-face with the autistic participant, either in a clinic setting or in their own home. **(See section on Data Collection on page 23.)**
2. *Presence of non-participants:* No non-participants were present during the interviews or focus groups.
3. *Description of sample:*

- The demographics of the professional practitioners are shown in Table 1 **(page 25)** including their age, gender, professional role, highest level of education, geographical location, years of experience, work context (e.g., individual therapy or school-based team), experience in assessing sensory processing, and number of clients with whom MYSET^®^ has been used.
- The demographics of the autistic participants are shown in Table 2 **(page 26)** including their age, gender, qualifications of the professional who diagnosed their autism, highest level of education, type of school attended, geographical location, language spoken at home, co-occurring conditions, level of social impairment measured by the *Social Responsiveness Scale* (2nd ed.), level of adaptive skills measured by the *Adaptive Behaviour Assessment System* (3rd ed.) The estimated IQ measured by the *Kaufman Brief Intelligence Test* (2nd ed.) was administered to three autistic participants. The IQ of the remaining two participants was assumed to be above average because of their high level of academic achievement (PhD and masters qualifications).
- The four family member participants (three mothers and one father) were aged 41-45 years; were all tertiary educated and spoke English at home **(see page 22).**

1. **Data collection**
2. *Interview guide:* The semi-structured interview and focus group questions are provided in Supplementary File C. The questions addressed perceptions of the extent to which the tool supports the autistic person to share their sensory experiences, the importance of others understanding their sensory experiences, and the role of the professional practitioner. Feedback was also gathered on usability issues including ease-of-use, length, comprehensiveness, the Impact Rating Scale, the strategy and support planning process, the cards, and card-sorting process. Feedback on the questions was sought from the first interview and the first focus group. No changes were recommended.
3. *Repeat interviews:* There were no repeat interviews.
4. *Audio/visual recording:* The interviews were video recorded. The video-recording software provides written transcript. These transcripts were all reviewed and corrected for errors in the transcription. **(See section on data collection on page 23.)**
5. *Field notes:* Field notes were taken on the interviewer’s impressions of the emotional responses of the interviewees.
6. *Duration:* The interviews ranged in duration from 25-70 minutes (autistic participants) and 30-70 minutes (family members).**. (See section on data collection on page 23.)**
7. *Data saturation:* The interviews and focus groups captured the perspectives of a broad range of professional practitioners, autistic participants and family members. As no new categories or sub-categories emerged in the later interviews and focus groups, we judged that the point of data saturation had been reached. **(See section on data analysis on page 27.)**
8. *Transcripts returned:* The transcripts were not returned, but one autistic participant, one family member and one professional practitioner reviewed the synthesized and analysed data and agreed that the way that their comments had been coded categories accurately represented their experiences.

**Domain 3: Data analysis and findings**

**Data analysis**

1. *Number of data coders:* Two coders independently coded 100% of the data. Using a consensus coding method. JA and JH met to review, discuss and collaboratively refine the codes until they reached agreement on the final codes (Cascio et al. 2019).
2. *Description of the coding tree:* Codes were organised into 6 categories and 3 sub-categories within Category 1, which are illustrated graphically in Figure 4. A coding tree (codes, code descriptors, sub-categories and categories) is provided at the end of this document in Supplementary File S2 Table 1.
3. *Derivation of themes:* Categories and sub-categories were derived from the data. Inductive content analysis was considered appropriate because this study aimed to explore specific and practical perspectives on using the tool in clinical practice without imposing predefined categories (Vears & Gillam, 2022).
4. *Software:* The data was coded manually.
5. *Participant checking:* One autistic person (AP01), one family member (FM04) and one professional practitioner (PP08) reviewed the synthesised and analysed data. The member checkers agreed that the way that their comments had been coded accurately represented their experiences. **(See section on data analysis on page 27.)**

**Reporting**

1. *Quotations presented*: Participants were assigned identifier numbers corresponding to their participant group (autistic participants = AP, family member = FM, professional practitioner = PP). Participant quotes are used to illustrate each subcategory.
2. *Data and Findings consistent*: Multiple participant quotes supported each of the sub-categories and these findings were reviewed by both coders to ensure consistency.
3. *Clarity of major themes:* Major six categories are clearly presented in Figure 4 including (1) MYSET^®^ key features, (2) Client’s self-reflection on sensory experiences, (3) Others’ understanding of client’s sensory experiences, (4) Customized reports on effective strategies and support planning, (5) Limitations to be addressed, and (6) Suggested improvements **(see page 29)**.
4. *Clarity of minor themes:* Each of the subcategories is clearly defined in Figure 4 as well as in the text. The subcategories under MYSET^®^ key features include (a) Nature of information gathered, (b) Accessibility to a diversity of autistic people and (c) Processes. There are clear descriptions of a diversity of perspectives. For example, some participants said that self-reflection through the use of MYSET^®^ enhanced understanding of their own sensory processing while others said that they already have good insight **(see page 29)**.

| **Supplementary File S2 Table 1:** Codes, code descriptors, sub-categories and categories | | |
| --- | --- | --- |
| **Codes and code descriptor** | **Sub-category** | **Category** |
| **Qualitative information gained through conversations**  Participants appreciated qualitative /narrative information gained through conversation | **Sub-category (1): Gathers individualised in-depth qualitative information** | **Category 1:**  **MYSET® key features** |
| **Pictures stimulate conversations**  Pictures prompted memories/ discussion about sensory experiences, uses visual strengths |  |  |
| **Comprehensive**  Nothing to be added or eliminated, time to gather comprehensive information is justified |  |  |
| **Captures autistic voice**  Provides a platform to express what is important and validates experiences |  |  |
| **Links directly to participation**  Easier to interpret than standardised tools due to focus on things that impact participation |  |  |
| **Gathers contextual information**  Important to discuss context, often “it depends” |  |  |
| **Collaborative process**  Collaborative therapeutic process, professional practitioner co-creates strategies with practitioner |  |  |
| **Young children**  Engaged young children, younger children understood process | **Sub-category (2): Accessibility to a diversity of people** |  |
| **People with mild intellectual disability**  People with mild intellectual disability were engaged and able to attend to it |  |  |
| **People with average/high IQ**  People with average/high IQ benefitted from using a tool with pictures. |  |  |
| **Proxy reporting option for people with high support needs**  Proxy reporting used with clients with high support needs, important to know person well, use multiple informants |  |  |
| **Ease of use**  Easy to follow instructions, professional facilitator provided clarification where necessary | ***Sub-category (3):* Processes** |  |
| **Flexibility**  Allows clients to participate in the way that that works for them, gives more options. |  |  |
| **4 step process**   1. What to expect stories supported understanding 2. Cards easy to understand due to combination of pictures and words 3. Impact Rating scale was beneficial 4. Strategy planning and support– planning was easy to use and helpful |  |  |

| **Codes and code descriptor** | **Category** |
| --- | --- |
| **Self-reflection enhances client’s understanding of their own sensory processing**  Self-reflection enhances understanding – thinking about what impacts them and how much it impacts them | **Category 2:**  **Client’s own understanding of sensory experiences** |
| **Some clients already have good insight**  Already aware of own sensory responses after years of self-reflection |  |
| **Enhances communication with other’s about sensory experiences**  Makes it easier to explain sensory experiences to others, others gained new insights | **Category 3:**  **Other’s understanding of client’s sensory experiences** |
| **Collation of new and existing strategies in a report customized to the person’s everyday environments**  Strategies were collated in a customised report to support participation in everyday environments such as their school or workplace. | **Category 4: Customized reports on effective strategies and support planning** |
| **Some people already have strategies while others need new strategies**  Some participants talked about strategies that they already use, as well as the need to develop new strategies |  |
| **Some strategies are used unconsciously**  People became more aware of strategies that that they already used unconsciously. |  |
| **Length challenging for younger children**  Too long for younger children - needs to be done over multiple sessions. | **Category 5: Limitations to be addressed** |
| **Clarity of *Impact Rating Scale***  *Impact Rating Scale* could be unclear due to need to differentiate the functional impact, emotional impact and effectiveness of management strategies. |  |
| **Confusion about wording on some cards**  Some younger participants found wording confusing due to number of words on the card or literal interpretation |  |
| **Participant stress due to indecision**  Some participants were anxious about making decision, and took a long time to sort each card |  |
| **Use of video-modelling to explain process**  Participants suggested that video-modelling may further enhance the explanatory process | **Category 6:**  **Suggested improvements** |
| **Some cards changed to reflect participant feedback**  Some participants suggested some additional cards. |  |
