# Supplementary material for: “My Sensory Experiences Tool”: A Neurodiversity‐Affirming Therapeutic Tool to Support the Sensory Challenges and Preferences of Autistic Children and Adults
Source: Occup Ther Int. 2026 Feb 25;2026:4779496. doi: 10.1155/oti/4779496 (PMC12933635; doi:10.1155/oti/4779496)
Supplement: Supplementary file 3 — Supporting Information 3 Supporting Information S3 contains the checklist used by observers to conduct fidelity observations of the MYSET sessions of the autistic participants. They observed that (1) clear concrete language was used to introduce MYSET, (2) the participants led the card sort, (3) the participants were offered the opportunity to ask questions, (4) the use of the ‘stop’, ‘take a break’, and ‘I have a question/I’m confused’ cards was explained to the participants, (5) the Impact Rating Scale was used by participants to rate the impact of their sensory experiences and (6) the participants’ own words were used on the Strategy and Support Planning and Review sheets. [file OTI-2026-4779496-s003.docx]

**Supplementary file S3: Fidelity checklist**

The fidelity checklist was designed by the research team and was included in the original project protocol that was submitted to a Human Research Ethics Committee. It was designed to ensure that the tool was administered as intended, in that it was underpinned by two key principles.

The first key principle was that the MYSET^®^ process was participant-led and under the participant’s control. This was achieved by ensuring that the participant led the card-sort. They could further control the process by using the “stop” or “take a break” card, if they wanted to halt or pause the process at any time. The participants were supported to communicate on the level of impact that their sensory experiences have on their life through the use of the ‘*Impact Rating scale*’. Additionally, the participants’ own words were used on the *Strategy and Support Planning and Review* sheet.

The second key principle was that there was clear communication between the participant and the professional practitioner. This was achieved through the use of clear concrete language and by inviting the participant to ask questions if unsure.

The research team members alternated between three roles, with one responsible for administering MYSET^®^, another observing the fidelity of the process, and another responsible for conducting the interview about the participant’s perception of MYSET^®^. The interviewing was done by the researchers with previous research experience (JA, VT and CD). The observations were either made in-person, or by observing videos of the MYSET^®^ being administered.

| **Fidelity checklist for administration of MYSET™** | | |
| --- | --- | --- |
| **Required** | | **Tick when observed** |
| 1. | Clear concrete language was used to introduce the MYSET^®^. |  |
| 2. | The participants led the card sort. |  |
| 3. | The participants were offered the opportunity to ask questions. |  |
| 4. | The use of the ‘stop’, ‘take a break’, and ‘I have a question/I’m confused’ cards was explained to the participants. |  |
| 5. | The ‘*Impact Rating scale’* was used to enable the participants to communicate the sensory experiences that have the biggest impact on their life. |  |
| 6. | The impact of the participant’s sensory experiences was documented in the participants’ own words on the *Strategy and Support Planning and Review* sheet. |  |
