# Supplementary material for: “My Sensory Experiences Tool”: A Neurodiversity‐Affirming Therapeutic Tool to Support the Sensory Challenges and Preferences of Autistic Children and Adults
Source: Occup Ther Int. 2026 Feb 25;2026:4779496. doi: 10.1155/oti/4779496 (PMC12933635; doi:10.1155/oti/4779496)
Supplement: Supplementary file 4 — Supporting Information 4 Supporting Information S4 includes the semi‐structured interview questions that were used with the autistic participants and family members, and the focus group questions that were used with the professional practitioners or therapy students. The questions covered the participants’ overall impression of the MYSET, ease of understanding of the cards and processes, the comprehensiveness of the cards, helpfulness of the tool in enabling others to understand the person’s sensory experiences, and helpfulness for generating strategies and accommodations that are compatible with their lifestyle. [file OTI-2026-4779496-s004.docx]

**Supplementary file S4**

**Semi-structured interview and focus group questions**

**Semi-structured interview questions for autistic participants**

- What did you think about using MYSET™? Did it help you share about your sensory experiences?
- How important is it to you that other people understand your own sensory experiences?

How useful/helpful was the tool in getting you to *think* about your sensory experiences?

How useful was the tool in helping you to *share/talk* about your sensory experiences?

How useful was the tool in helping identify the main sensory experiences that you would like support with?

- Do you feel that this tool would help you to talk about your sensory experiences with someone else (i.e., teacher/therapist/psychologist/planner/employer)?
- Did you have a professional (e.g., teacher/therapist/psychologist) support you to complete MYSET™?
- If no, how easy was it to complete without professional support?
- Did you have a trusted adult (e.g., family member or carer) support you to use the MYSET™?
  - If yes, what did they do to help you use MYSET™?
- Who supported you in using MYSET™ (i.e., a parent, teacher, therapist, psychologist etc.)?
- Did you already know this person and feel comfortable with them before doing MYSET™?
- How important do you think it is that the person supporting you to use MYSET™ is someone you know and feel comfortable with?
- Tell me about how you found using MYSET™.
- How easy was it to use? Were the instructions easy to follow?
- How long did it take? How did you find the length/number of cards?
- How easy was it to use the rating scales? Were these useful?
- How easy was it to think about strategies and have these written on the plan? Was this helpful?
- Tell me a bit about what you thought of the actual cards.
  - Were the words on the cards straight forward and easy to understand?
  - Did the words help you to think about your sensory experiences?
  - Did the pictures make it easier for you to think about your sensory experiences? If so, why do you think that was?
- Do you think the tool covers the range of sensory experiences that are likely to be important to some autistic people?
  - Can you think of any other sensory experiences that were missing and that you think should be included?
- While using the tool, did you come across anything that you found confusing or that you think needs to be changed (i.e., cards, rating scales, sorting plates)?
- Why/how was it confusing?
- What changes do you think need to be made?
- Are there any goal cards you think should be taken out?
- Did someone need to explain any of the cards to you?
- Were the rating scales easy to use?
- Can you think of any changes to how you use the tool that would make it easier/better?
- Tell me a bit about other times you have shared information about your sensory experiences.
- Who did you share the information with?
- Have you done a sensory processing assessment before? Did you use a tool?
- How is this tool different to those other times? Which way of sharing about your sensory experiences do you like better?
- Would you use MYSET™ again, or tell other people that it is good to use?
- What do you think about the tool being offered electronically (i.e., on an iPad, etc.) rather than having physical goal cards that you can hold?

**MYSET™ Semi-structured interview questions for family members**

- How involved were you in using the tool with your autistic family member?
- Tell me a bit about your experience of the process of using MYSET™.
- How easy was it to use? Were the instructions easy to follow?
- How long did it take? How did you find the length/number of cards sort?
- How easy was it to use the rating scales?
- How easy was it to think about strategies and have these written on the plan? Was this helpful?
- Did you have professional support to complete MYSET™?
  - If no, how easy was it to complete without professional support?
- How useful was the tool in helping you to identify and prioritise sensory experiences that your autistic family member would like support with?
- Do you feel that this tool would help you and/or your autistic family member to communicate their sensory experiences to someone else (i.e., teacher/psychologist/therapist/planner/employer)?
- Tell me a bit about what you thought of the cards.
  - Were the words on the cards straight forward and easy to understand? Do you think the wording on each card captured the sensory experience?
  - Did the words help you and your autistic family member to reflect on/think about their sensory experiences?
  - Did the pictures make it easier for you and your autistic family member to reflect on/think about their sensory experiences? If yes, why do you think that was?
- Do you think the tool adequately captures the range of sensory experiences that are likely to be relevant for some autistic people?
- Can you think of any additional sensory experiences that you felt were missing and that you would recommend including?
- While using the tool, did you come across anything that you found confusing or that you think needs to be changed (i.e., cards, rating scales, sorting plates)?
  - Were there parts of the tool that you needed to spend more time explaining to your autistic family member?
  - Why/how was it confusing?
  - What changes would you recommend?
  - Would you recommend removing any cards?
- Can you suggest any changes to the process of using the tool that you think would improve it?
- Tell me a bit about your previous experiences with sharing information about your autistic family member’s sensory processing.
  - Who were you sharing the information with?
  - Have you or your autistic family member shared information about their sensory experiences before? Did you/they use a tool?
  - How does MYSET™ compare to other methods? Which method do you prefer?
- Would you consider using this tool in the future, or recommend it to other people?
- What are your thoughts on the tool being offered electronically (i.e., on an iPad, etc.) rather than having physical cards that you can hold?

**MYSET™ Focus group questions for professional practitioners/therapy students**

- Tell me a bit about your overall impression of MYSET™.
- How easy was it for you to use? Are the instructions easy to follow?
- How long did it take? How did you find the length/number of cards sort?
- How easy was it to use the rating scales?
- How easy was it to think about strategies and have these written on the plan? Do you think this was helpful?
- How useful do you think the tool will be in helping autistic people to identify and prioritise sensory experiences that they would like support with?
- How useful do you think the tool will be in helping your autistic clients to communicate about their sensory experiences?
- How comfortable would you feel about supporting your clients with the sensory experiences they identified as having a big impact on their life?
- Tell me a bit about what you thought of the cards.
  - Are the words on the cards straight forward and easy to understand?
  - Do you think the wording on each card captures the sensory experience?
  - Do the pictures accurately reflect the sensory experience?
- Do you think the tool adequately captures the range and types of sensory experiences that are likely to be relevant for some autistic people?
- Can you think of any additional sensory experiences that you feel are missing from the card-sort and that you would recommend including?
- While going through the tool, did you come across any aspects of the tool that you found confusing or that you think need to be changed?
- Were there aspects that required additional explanation for your client?
- Why/how was it confusing?
- What changes would you recommend?
- Would you recommend removing any cards?
- Can you suggest any changes to the process of using the tool that you think would improve it?
- Tell me a bit about your current/usual methods of gathering information about/assessing a client’s sensory experiences/sensory processing.
  - How does this tool compare to other methods? Which method do you prefer?
  - What are the benefits/limitations of each method?
- Would you consider using this tool again in the future, or recommend it to people?
- What are your thoughts on the tool being offered electronically (i.e., on an iPad, etc.) rather than having physical cards that you can hold?
